# High Rates of Obesity and Non-Communicable Diseases Predicted across Latin America

**DOI:** 10.1371/journal.pone.0039589

**Published:** 2012-08-13

**Authors:** Laura Webber, Fanny Kilpi, Tim Marsh, Ketevan Rtveladze, Martin Brown, Klim McPherson

**Affiliations:** 1 National Heart Forum, London, England; 2 New College, University of Oxford, Oxford, England; Fundación para la Prevención y el Control de las Enfermedades Crónicas No Transmisibles en América Latina (FunPRECAL), Argentina

## Abstract

Non-communicable diseases (NCDs) such as cardiovascular disease and stroke are a major public health concern across Latin America. A key modifiable risk factor for NCDs is overweight and obesity highlighting the need for policy to reduce prevalence rates and ameliorate rising levels of NCDs. A cross-sectional regression analysis was used to project BMI and related disease trends to 2050. We tested the extent to which interventions that decrease body mass index (BMI) have an effect upon the number of incidence cases avoided for each disease. Without intervention obesity trends will continue to rise across much of Latin America. Effective interventions are necessary if rates of obesity and related diseases are to be reduced.

## Introduction

Internationally the obesity epidemic is driving up the burden of several non-communicable diseases (NCDs) such as cancers, heart disease and diabetes. In Latin America, non-communicable diseases are amongst the biggest killers and rates of these diseases are expected to escalate. For example, diabetes is predicted to increase by more than 50% with 32.9 million suspected sufferers by 2030 in Latin America [1]. Overweight and obesity are key modifiable risk factors for NCDs and with rates as high as 60% amongst adults (Ministry of Health Belize, Ministerio de Salud Nicaragua, Ministry of Public Health, El Salvador) the epidemic is a major public health concern both to individual quality of life, longevity, and costs to health systems.

Across Latin America the obesity epidemic has been driven by the rapid demographic and nutritional transition as countries go through a changing economic climate and emerge from poverty [2], [3]. Demographically, populations of Latin America are ageing as there has been a shift from high to low fertility and mortality. Nutritionally, an increased intake of energy dense foods high in sugars and saturated fats coupled with increased inactivity levels are key factors explaining the rise in obesity. Urbanisation and economic growth has driven this change. Alongside this, sedentary lifestyles are commonplace with between 30–60% not meeting the recommended levels of physical activity each day [4] with a shift to industrialised cities and the loss of the protective rural aboriginal environment [5]. The highest levels of obesity are seen in urban poor women, though they affect both genders. The World Health Organisation has responded to the increasing burden of NCDs by putting in place global strategies such as that for the prevention and control of chronic diseases. Aims have been made to increase surveillance, improve public awareness and facilitate quality of care for chronically ill patients [6]. Though there is a long way to go and greater prevention strategies.

Knowing the direction and speed of change of obesity rates is necessary if health policies that aim to reduce obesity are well-placed and effective. This study used microsimulation modelling to project obesity trends and related burden of disease in Latin America to 2050 using the data available.

## Methods

### Data sources

BMI data was collected by reviewing the literature using Pubmed (supplemented by Google scholar). Unpublished data was collected through personal communication with researchers and authors of published studies. The WHO BMI database was used as a further source of BMI data and references. Articles were included if they contained BMI data presented by age and sex (see Table S1 for a table of references used for BMI data). Because of the scarcity of data, sub-national and national, measured and self-reported data were included.

A second review of the literature was carried out to locate country-specific incidence, survival and mortality rates of obesity-related diseases – type 2 diabetes, coronary heart disease, stroke and obesity-related cancers (colorectal, pancreas, breast, kidney, liver, endometrial and oesophageal).

### Extrapolation of missing data

Few countries had more than two data points and three countries – Costa Rica, Cuba, Panama - only had one. For these countries 2008 estimates were used based on a recent analysis by Finucane and colleagues [7]. This extrapolates from their estimated mean: the BMI-distribution is assumed to have the form {p,(1-p)/2,(1-p/2)} where p is the prevalence of normal weight; p is then determined from the known mean.

For Bolivia, Nicaragua and Peru only data on females was available.

### Data manipulation

It was necessary to manipulate the BMI data in a number of ways: We sorted the source data into three mutually exclusive BMI categories: normal weight (<25 kg/m^2^), overweight (25–29.9 kg/m^2^), and obese (≥30 kg/m^2^). Where some data were in wide age groups (e.g. 20 year age groups) they were divided into 10-year and 5-year age groups, doubling or quadrupling the variance of the estimates as appropriate. Variance was calculated using the equation (p(1-p)/n) where: n is the sample size and p the prevalence.

### Proxy country data

Where disease data were not available then data from a proxy country were used instead. Proxy countries were chosen based on the proximity and similarity to the target country. For fatal diseases (coronary heart disease, stroke, cancers) a proxy country's incidence data was adjusted using the, known, target country's mortality rate: the ratio of the target-to-proxy countries' mortality rates was used to scale the proxy country's incidence rates. To estimate incidence rates from proxy countries for non-fatal diseases (e.g. Type II diabetes the proxy country's BMI, relative incidence rate statistics and population statistics are used to determine the incidence rate for people having normal BMI in the proxy country. In the target country, its own BMI statistics are used to estimate the incidence stats (the relative risk statistics are assumed to be universal). For fatal diseases target country death rates were also included in the calculation.

For coronary heart disease, Mexico was used as a proxy for all Latin American countries. UK CHD incidence figures were adjusted for the difference in CHD mortality between the UK and each target country using the WHO Global Infobase. This was done by scaling the UK incidence figures by the ratio of the age standardised mortality rate in 2008 to the same figure for the UK [8]. The idea is that mortality/incidence is approximately constant so that, suppose in the Target country mortality is known but incidence is not known, then incidence_T_ = (incidence_P_/mortality_P_)mortality_T_ [T = target P = proxy]. The same database was also used for CHD mortality rates. Survival data from the US was used as a proxy [9].

For stroke, Chile was used as a proxy for all Latin America countries adjusted for the difference in CHD mortality for each target country by the method described. Stroke survival was taken from US figures [10].

Mexico was the only country with appropriate incidence data for diabetes [8] and so this was used as a proxy for all other Latin American countries.

Globocan 2008 [11] was used for incidence and mortality rates in Latin America. Survival rates for cancers were taken from Costa Rica for breast cancer [12] Cuba for colorectal and kidney cancer [13], [14], Brazil for endometrial [15], Puerto Rico for Liver cancer and the US for Oesophagus and Pancreatic cancer [16]. A table of disease references for each country is presented in Table S2. Incidence, mortality and survival input data are presented in Table S3.

### Calculation of survival

For survival data, the probability of survival, p, for a number of years, T, after acquiring a fatal disease was modelled in one of two ways depending on the disease. Either as a simple exponential distribution p = e^−RT^, or as an exponential distribution allowing for different probability, p_1_, of survival in the first year, p = p_1_e^−R(T-1)^. Stroke used the latter model; other fatal diseases the former. Disease survival statistics consist of the rate R or the rate R together with the first year survival probability p_1_. These statistics are further classified by age group and gender. The rate R was usually inferred from quoted 5-year survival statistics.

For coronary heart disease, Mexico was used as a proxy for all Latin American countries. Incidence statistics were adjusted for the difference in CHD mortality for each target country [8] and the UK by scaling the UK incidence figures by the ratio of the age standardised mortality rate in 2008 to the same figure for the UK [8]. The same database was also used for CHD mortality rates. Survival data from the US was used as a proxy [9].

### Statistical analysis

BMI trends and future obesity-related disease burdens were estimated to 2050 by age and sex for 10 Latin American countries using micro-simulation modelling. This method is described in greater detail in Wang and colleagues [17] and Methods S1 of the supplementary information. A dual-module modelling process was developed by the UK Foresight working group [18], [19] which was applied and refined for this study. Module one uses a non-linear multivariate, categorical regression model fitted to cross-sectional BMI data from each of the countries. Module two uses a micro-simulation program to produce longitudinal projections to 2050. This creates a virtual population cohort based on module one BMI distributions from 2010 to 2050. A BMI value is probabilistically assigned as a function of age, sex and calendar year. BMI trajectories were projected using the simulation model with the assumption that an individuals' BMI percentile in the same age cohort stays the same over time. Population size, births and deaths were also simulated in a large number of individuals as they age using data from the World Health Organization and United Nations. Simulated individuals are at risk of getting a particular disease each year if he or she did not have the disease at the beginning of the year; they can continue living with the disease or die from it (if it is fatal). The software for this program was written in C++. Outliers were removed. One million Monte Carlo runs per country were carried out, but due to the scarcity of some if BMI data, running a whole population might have resulted in smoother curves. To estimate the disease burden associated with the trends in overweight and obesity, as well as the effect of possible interventions, future increases in obesity-related diseases were projected from 2008 to 2050, using three different trend scenarios: scenario 0: obesity trends go unchecked; scenario 1: obesity levels decrease by 1% and scenario 2: obesity levels decrease by 5%.

## Results

The results presented below were simulated from three separate scenarios which differed in the environment assumed to prevail for the period 2000 to 2050.

- Scenario 0: 2000 to 2050; unrestricted BMI growth as projected
- Scenario 1: 2000 to 2050; 1% BMI reduction relative to scenario 0
- Scenario 2: 2000 to 2050; 5% BMI reduction relative to scenario 0

**Figure 1:**
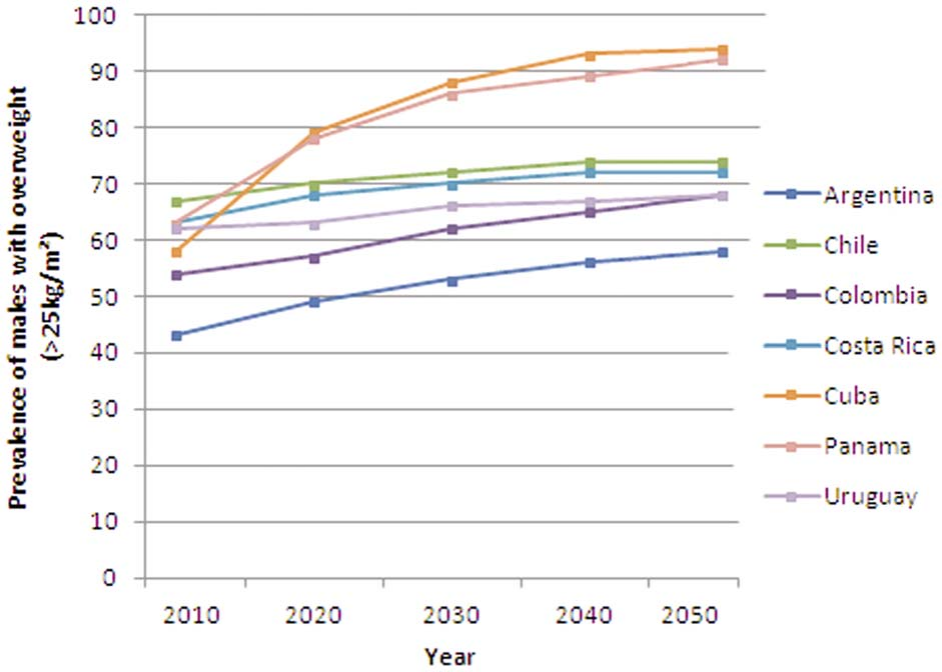
Past and projected prevalence of obesity in Latin American males (BMI ≥25 kg/m2) based on module 1, scenario 0.

**Figure 2:**
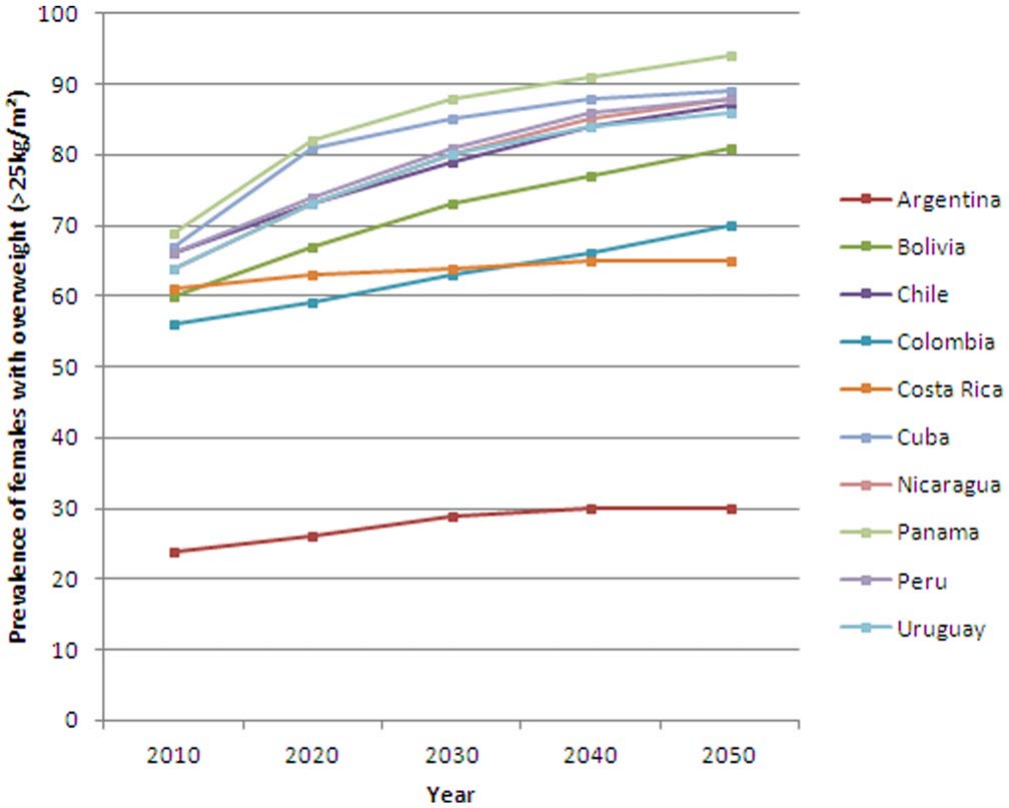
Past and projected prevalence of obesity in Latin American females (BMI ≥25 kg/m2) based on module 1, scenario 0.

Figure 1 and Figure 2 shows projected prevalence of overweight (≥25 kg/m^2^) in males and females respectively aged 20+ years. Across all countries overweight and obesity are projected to increase by 2050 in both males and females. In females, much lower levels were seen in Argentina than other countries. However, since only two sub-national data points were available, interpretation of this result should be made with caution. By 2030 more than 50% of males and 60% of females (excluding Argentina) will be overweight or obese. The highest projected rates were seen in Cuba and Panama. Table S4 and S5 presents the projected percentage of overweight and obese males and females respectively.

**Figure 3:**
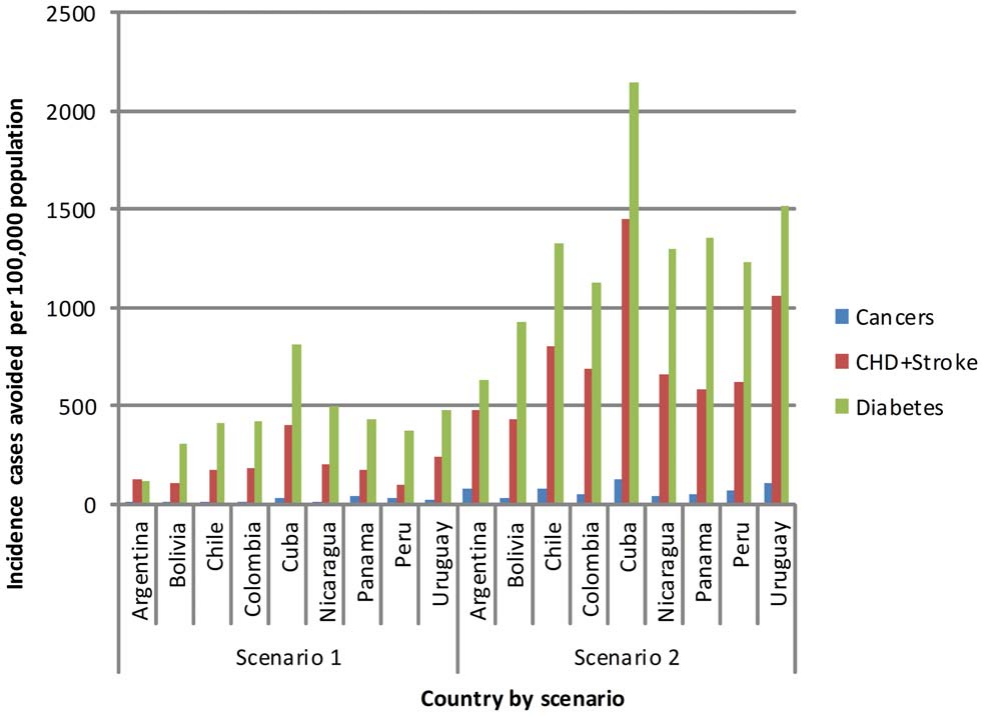
Cumulative incidence cases avoided of cancer, CHD+stroke and diabetes per 100,000 population across Latin America by 2030.

Figure 3 presents the cumulative incidence cases avoided of cancer, CHD & stroke, diabetes per 100,000 population across Latin America by 2030. Effective interventions that reduce obesity levels will have a dramatic effect upon the cumulative incidence cases avoided. The biggest impact would be in Cuba where more than 1300 cases of CHD and stroke and more than 2000 cases of type 2 diabetes are avoided per 100,000 of the population with a 5% reduction in BMI. Based on total population figures (UN population data 2011), the number of cases of CHD & Stroke in Cuba will rise to over 1340000, cases of diabetes to 1030000, and cases or cancer to 220000 by 2030 in the total adult population (aged 20+ years). To use two other examples, in Colombia, 175000 people will have CHD & Stroke, 149147 will have diabetes and 35300 will have cancer in the total population by 2030. In Uruguay, 340000 will have CHD&Stroke, 207000 will have diabetes, 76000 will have cancer by 2030. A table of cumulative incidence cases avoided for 2030 is presented in table S6. It is important to note that disease projections for Bolivia, Peru and Nicaragua are based on female only BMI data.

## Discussion

Using sophisticated modelling techniques the results of this study illustrated how the obesity epidemic will unfold across Latin America. Over the next twenty years overweight and obesity was projected to increase. Reflecting these trends the incidence of each disease is also set to increase. Interventions that are effective in reducing BMI will be important in reducing rates of cardiovascular disease and diabetes.

In general, overweight and obesity levels were projected to increase in all countries with the highest rates seen in Cuba. Rates of change are likely to be a result, at least in some part, to economic changes. The positive effect of the economic transition has been to help eradicate undernutrition, but, unregulated, it also promotes unhealthy lifestyles which favour obesity. Traditional diets in the region are often meat-based, in particular red meats are popular staples and food preparation involving frying is also popular. When food security and incomes increase, it is not surprising that the per-capita consumption of these traditional foods increase, together with other Western influences on diets. Chile had the best available data of any Latin American country allowing for more accurate predictions than other countries. In the 1990s Chile doubled its per capita income. An important amount of this increase has been spent on modern living such as television sets, cars and unhealthy high fat, sugar and salted processed foods fuelling energy imbalance and subsequent increases in obesity [20]. Similar transition has occurred across Latin America.

Cuba is an interesting case due to the macroeconomic changes and their consequences on health behaviour and obesity. Since the 1950s the economy grew at a rate higher than the rest of Latin America despite the US embargo. The collapse of the Soviet Union in the 1990s cut Cuban trade by 80% and GDP decreased by 33% [21] resulting in food shortages, increased physical activity and reduced BMI [22]. However, the budget for education and health increased during this period. Since then, availability of high fat foods has increased, whereas labour-intensive activity has reduced, which may be one contributing factor of the plethora of causes explaining the obesity epidemic and the high levels of obesity and cardiovascular diseases observed in the present study.

Cumulatively, from 2010 to 2030 the incidence rate of CHD & Stroke in Cuba would rise to a staggering 15022 per 100,000 of the population, diabetes would rise to 11624, and cancer to 2523 per 100,000 of the population. Cuba has a good quality health system relative to the rest of Latin America. That the current estimates of Cuba's CHD rates are higher than elsewhere in Latin America might be a result of higher survival rates due to better quality health care.

As well as economic changes, socio-economic and demographic differences between and within countries are likely to impact the rate of obesity. Obesity is shifting to be a disease of the poor, as it is in most high-income countries [23] which underscores obesity as a social phenomenon highlighting the need to take action on sociocultural and economic factors. This social shift in obesity usually happens first in urban women perhaps because of differences in working patterns between men and women with men being in more traditionally manual labour roles. A study by PAHO/WHO [6] on obesity in Latin America found that a higher prevalence of obesity is correlated with per capita income especially in urban areas. Despite this, under nutrition is still a major problem and it is increasingly apparent that Latin America is experiencing a double burden of disease where both underweight and obesity coexist.

Interestingly, more urban countries show a higher rate of disease than less urban countries. For example, in highly urbanised Chile the cumulative incidence rate of CHD and stroke by 2030 is around 8100 per 100,000 of the population in 2010 [24]. In Nicaragua, where urbanisation has reached 58%, the projected cumulative incidence (for women) is 5400 by 2050 per 100,000 of the population in 2010. There is also a huge ethnic diversity within and between countries in Latin America. Interestingly, nations with the highest white or European ethnicities and that are highly urbanised (e.g. Uruguay, Chile) have the highest projected prevalence rate of cardiovascular diseases compared with countries such as Bolivia, Colombia and Nicaragua where the ethnic makeup is mostly Amerindian and/or Mestizo. However, in Bolivia and Nicaragua we were only exploring women who have a lower risk of CVD than men especially in the younger age groups and these populations are still quite young. Sampling both men and women will allow for more accurate comparisons to be made.

The results of this study have important policy implications. Given the high social and economic cost of NCDs, further work into the health economics of obesity in Latin America is necessary so that future health policy can be planned for. In 2000, diabetes was estimated at US$65.2 billion across Latin America [25] and a recent review reported that obesity accounted for 0.7–2.8% of a country's total health care costs and medical costs were 30% higher for obese than normal weights [26]. Thus the problem of obesity poses an enormous challenge and institutionally Latin America needs to be equipped to deal with increasing numbers of chronic diseases. Some countries have responded to the obesity challenge by implementing interventions. Chile introduced nutrition and physical activity initiatives to reduce obesity in preschool children though this was not enough to shift the rising rate of obesity. Although, it was argued that the intervention's lack of success is perhaps because obesity rates have reached a plateau [27]. To address the problem of sedentary lifestyles, Colombia has launched a free bike scheme and bicycle lanes in the capital Bogota which has since been named the worlds 3^rd^ most bike-friendly city. The Caribbean Public Health Agency (CARPHA) [28] have recently set non-communicable diseases as a key public health priority (2011) and in early 2011 more than 40 Latin American organisations launched The Healthy Latin American Coalition (HLAC) to develop a declaration recognising the public health emergency of NCDs and the importance of government action. Clearly, if trends are set to continue rising more work is required.

Very little data were available for Latin American countries making analysis of obesity trends in this area limited and it difficult to draw affirmative conclusions. For Bolivia, Nicaragua and Peru data for females only was available. These data were from Demographic Health Surveys which only measure women who had had a child in the past five years, thus biasing the data. The direction of the bias is unclear, and might differ according to age, but higher rates of overweight might be expected since mothers often do not return to pre-pregnancy weight. For Costa Rica, Cuba and Panama only one data point was available and so 2008 estimates were used based on a recent analysis by Finucane and colleagues [7] which used BMI means. This is disadvantageous since one cannot then reliably infer the proportion of obese to overweight. However the estimates are of use when looking at the proportion of normal weight to overweight and obese combined. This highlights the need for greater surveillance work across Latin America which samples both men and women in nationally representative samples. This is imperative if accurate estimates of trends are to be made and policies to be built around more precise data.

The projections can only be as good as the data that is input. Our extensive searches found very little data were available for Latin America and no set of complete age and sex-specific BMI and disease data for one Latin American country. We were also unable to include data on children due to lack of consistently measured data. Since projections are mere extrapolations from these data, inaccuracies in the output are likely. However, there was insufficient time to undertake time consuming error analysis. Furthermore, we have insufficient knowledge of BMI growth patterns following interventions and insufficient knowledge of the future.

Our model incorporates a sophisticated economic module using Morkov-type simulation estimation of long-term health benefits, health care costs and the cost-effectiveness of specified interventions. With access to country-specific cost data our model can be adapted to include cost burden and allow us to simulate costs of obesity-related diseases for application in public health policy. Our recent work projected future health and related medical costs based on available disease data in the UK and US [17] allowing for more accurate projections to be forecast, however these data were not available for Latin America and so could not be included in the present study. Other diseases beyond those studied here have been related to obesity such as infertility [29], sleep apnoea [30], osteoarthritis [31], asthma [32]. It was beyond the scope of this study to include them. The flexibility of our model means that there is scope to model these diseases given the right input data are made available.

The programme is limited in that it assumes that people do not reverse in the BMI categories. Unfortunately this mirrors reality, where body weight loss is often only temporary. Moreover, the analysis has not taken into account unforeseeable changes in circumstances, such as fluctuations in food prices and changes in medicine. It relies on our best estimate based on previous trends.

The 95% confidence intervals for the microsimulation were derived from simulation of the BMI distributions corresponding to the upper and lower limits of each of the obesity growth scenarios. Unfortunately there was insufficient time to undertake time consuming error analysis and this has been noted in the limitation of the paper. The results do not vary significantly when different simulations are run.

Despite some limitations, this study is timely and an important first step in quantifying the future burden of obesity-related diseases in Latin America. It highlights the need for urgent action to curb obesity levels and reduce the burden of disease. The challenge is to understand how best to initiate change and to quantify the cost of health consequences of obesity. If governments take action by implementing effective policies that reduce overweight and obesity, then a substantial number of new cases of cancer and cardiovascular diseases can be avoided in the coming decades.

## Supporting Information

Methods S1 **Statistical methods.**
(DOCX)

Table S1 **References used for BMI data in each country.**
(DOCX)

Table S2 **References used for each disease in each country.**
(DOCX)

Table S3 **Incidence, mortality and survival input data for each country.**
(DOCX)

Table S4 **Percentage of overweight and obese males in Latin America projected to 2050.**
(DOCX)

Table S5 **Percentage of overweight and obese females in Latin America projected to 2050.**
(DOCX)

Table S6 **Cumulative incidence cases avoided per 100,000 of the population in 2010 with a 1% (scenario 1) and 5% (scenario 2) decrease in body mass index by 2030.**
(DOCX)
