# Supplementary material for: High Rates of Obesity and Non-Communicable Diseases Predicted across Latin America
Source: PLoS One. 2012 Aug 13;7(8):e39589. doi: 10.1371/journal.pone.0039589 (PMC3418261; doi:10.1371/journal.pone.0039589)
Supplement: Methods S1 — Statistical methods. (DOCX) [file pone.0039589.s001.docx]

Methods S1: Statistical methods

An individual's body mass index (BMI) is defined as:

$BMI=\frac{w}{h^{2}}$,

where $w$ and $h$ correspond to individual's weight and height, respectively. $BMI$ provides a simple measure of a person's “fatness” or “thinness”. Although $BMI$ is measured on a continuous scale, it is grouped in the following five categories:

1. $BMI$: <18.5 (underweight)
2. $BMI$ from 18.5 to 24.99: (healthy weight)
3. $BMI$ from 25 to 19.99: (overweight)
4. $BMI$ from 30 to 39.99: (obese)
5. $BMI \geq40$: (morbidly obese)

Let $g$=1,...,5, denote $BMI$ group (lower and larger values of $g$ correspond to groups with lower and larger values of $BMI$ on the continuous scale, respectively). Let $q_{g}(t)$ be the prevalence of individuals with $BMI$ values that correspond to group $g$ at time $t$. To ensure that $q_{g}\left( t \right)$takes values within [0,1], we model $q_{g}\left( t \right)$by

$q_{g}(t)=\frac{1}{2}\left[ 1+\tanh\left( \beta_{0}^{g}+\beta_{1}^{g}t \right) \right].$ (1)

An alternative way to estimate $q_{g}(t)$ is through a logistic regression model with percentage for $BMI$ group $g$ as the outcome, and time, $t$, as the single explanatory variable:

$\ln\left( \frac{q_{g}(t)}{1-q_{g}(t)} \right)=\beta_{0}^{g}+\beta_{1}^{g}t.$ (2)

By solving equation (2) for $q_{g}(t)$we obtain

$$q_{g}\left( t \right)=\frac{\exp\left( \beta_{0}^{g}+\beta_{1}^{g}t \right)}{1+\exp\left( \beta_{0}^{g}+\beta_{1}^{g}t \right)}.$$

Equations (1) and (2) are mathematically identical, but (1) provides greater numerical stability. Fitting separate models for the estimation of $q_{g}(t)$ for each group g, does not guarantee that

$\sum_{g=1}^{5} q_{g}(t)= 1$.

Thus, to ensure that the estimates of prevalence of individuals in all $BMI$ groups sum up to 1 for each time $t$, we estimate the prevalence of individuals with $BMI$ values that correspond to group $g$ at time $t$ by

$P_{g}\left( t \right)=\frac{q_{g}\left( t \right)}{\sum_{g=1}^{5} q_{g}\left( t \right)}.$ (3)

**Micro simulation – BMI growth model**

The distribution of BMI in the population is estimated using regression analysis stratified by both sex (S) and age group (A=0-9, 10-19,20-29,…,70-79, 80+). The fitted models are extrapolated to forecast the distribution of BMI groups in the future. For each sex-and-age-group stratum, the set of cross-sectional, time-dependent, discrete distributions of BMI groups, $D=\{P_{g}\left( t \right)|g=1,\ldots5; t>0\}$, is used to manufacture BMI growth models for individual members of the population. This is done in a way that guarantees that the cross-sectional BMI group distributions obtained by simulation under the growth models match the BMI group distributions of the observed data. The details are as follows;

For each such discrete distribution in $D$, there is a continuous counterpart. Let $\beta$ denote BMI in the continuous scale and let $f(\beta|A,S,t)$ be the probability density function of $\beta$ for age group $A$ and sex $S$ at time $t$. Then

$P_{g}\left( t | A,S \right)=\int_{\beta\in g} f\left( \beta| A,S,t \right)d\beta.$ (4)

Equations (3) and (4) both refer to the same quantity. However, equation (4) uses the definition of a probability density function to express the age-and-sex-specific percentage of individuals in BMI group g at time t. Equation (3) gives an estimate of the this quantity using model (1) for all g=0,..,5. The cumulative distribution function of $\beta$ is

$F\left( \beta| A,S,t \right)=\int_{0}^{\beta} f\left( \beta| A,S,t \right)d\beta.$ (5)

At a time t, a person with sex $S$ belonging to the age group $A$ is said to be on the $p$–th percentile of this distribution if $F\left( \beta| A,S,t \right)=p/100.$ Given the cross-sectional information of $D$, it is possible to simulate longitudinal trajectories by forming pseudo cohorts within the population. A key requirement for these sets of longitudinal trajectories is that they reproduce the cross-sectional distribution of BMI groups for any year with available data. The method adopted here and in the earlier Foresight report^[[1]](#footnote-1)^ is based on the assumption that people’s BMI changes throughout their lives in such a way that they always stay on the same BMI percentile. As they age, individuals move from one age group to another and their BMI changes so that they remain on the same percentile but of a different distribution. This rule is not too far from the truth; and has as a result that relatively fat people stay relatively fat and relatively thin people stay relatively thin. Crucially it meets the important condition that the cross-sectional BMI group distributions obtained by simulation match the BMI group distributions of the observed data. This can be seen as follows:.

When the population’s BMI distributions by sex and age are known for all years (by extrapolation of fitted model (3)), a person who is in age group $A$ and who grows ten year older will at some time move into the next age group $A'$ and will have a BMI that was described first by the distribution $f(\beta|A,S,t)$ and then at the later time $t'$ by the distribution $f(\beta|A',S,t')$. If the BMI of that individual is on the p^th^ percentile of the BMI distribution, his BMI will change from $b$ to $b'$ so that

$\beta=F^{-1}\left( \frac{p}{100} | A,S,t \right)$ (6)

$\beta^{'}=F^{-1}\left( \frac{p}{100} | A^{'},S,t^{'} \right)\underset{\Rightarrow}{}\beta^{'}=F^{-1}\left( F(\beta|A,S,t) | A^{'},S,t^{'} \right)$ (7)

Where $F^{-1}$ is the inverse function of the cumulative distribution function of $\beta$. Equation (7) guarantees that the transformation taking the random variable $b$ to $b'$ ensures the correct cross-sectional distribution at $t'$.

The micro simulation both generates individuals from the BMI distributions of the set $D$ and, once generated, grows the individal’s BMI in a way that is also determined by the set $D$. It is possible to implement equation (7) as a suitably fast algorithm.

**Micro simulation: Birth, disease and death models**.

Simulated people are generated with the correct demographic statistics in the simulation’s start-year. In this year women are stochastically allocated the number and years of birth of their children – these are generated from known fertility and mother’s age at birth statistics (valid in the start-year). If a woman has children then those children are generated as members of the simulation in the appropriate birth year.

In the course of their lives, simulated people can die from one of the BMI related diseases that they might have acquired or from some other cause. The probabilities that a person of a given age and gender dies from a cause other than a BMI related disease are calculated in terms of known death and disease statistics valid in the start-year and are held constant over the course of the simulation. The death rates from BMI related diseases will change as a consequence of the population’s changing BMI distribution.

1. Foresight report [↑](#footnote-ref-1)
