# Supplementary material for: High Rates of Obesity and Non-Communicable Diseases Predicted across Latin America
Source: PLoS One. 2012 Aug 13;7(8):e39589. doi: 10.1371/journal.pone.0039589 (PMC3418261; doi:10.1371/journal.pone.0039589)
Supplement: Table S1 — References used for BMI data in each country. (DOCX) [file pone.0039589.s002.docx]

Table S1 References used for BMI data in each country

| Country | Study name citation | Data year(s) | Sample size | | Agegroup | Measured/self-reported | National/regional data |
| --- | --- | --- | --- | --- | --- | --- | --- |
| **Argentina** |  |  | **M** | **F** |  |  |  |
|  | Carbajal et al, 2001 | 1998 | 556 | 967 | 15-75 | Measured | Subnational |
|  | WHO: Virgolini and Ferrante (no date given) | 2003 | 1100 | | 15-75 | Measured & self-report | Subnational |
| **Bolivia** |  |  |  |  |  |  |  |
|  | WHO; Encuesta Nacional de Demografia y Salud 1998 | 1998 | - | 3744 | 15-49 | Measured | National |
|  | DHS | 2003 | - | 16075 | 15-49 | Measured | National |
|  | DHS | 2008 | - | 20786 | 15-49 | Measured | National |
| **Chile** |  |  |  |  |  |  |  |
|  | Encuesta Nacional de Salud | 2003 | 1559 | 1869 | 17-100 | Measured | National |
|  | Encuesta Nacional de Salud | 2010 | 1977 | 2931 | 15-65+ | Measured | National |
| **Colombia** |  |  |  |  |  |  |  |
|  | DHS | 1995 | - | 3156 | 15-49 | Measured | National |
|  | DHS | 2000 | - | 3069 | 15-49 | Measured | National |
|  | DHS | 2005 | - | 33244 | 15-49 | Measured | National |
|  | DHS | 2010 | - | 43725 | 15-49 | Measured | National |
| **Costa Rica** |  |  |  |  |  |  |  |
|  | CARMEN, Ministerio de Salud | 2001 | 393 | 531 | 20-64 | Measured | Subnational |
|  | Encuesta Multinacional de Diabetes mellitus y Factores de Riesgo | 2004 | 509 | 882 | 20-65+ | Measured | Subnational |
| **Cuba** |  |  |  |  |  |  |  |
|  | Acosta et al 2005, Estado nutricional de la poblacion cubana adulta | 2002 | 19519 | | 17-100 | Measured | National |
| **Nicaragua** |  |  |  |  |  |  |  |
|  | DHS | 1998 | - | 4793 | 15-49 | Measured | National |
|  | DHS | 2001 | - | 11836 | 15-49 | Measured | National |
|  | WHO: data of SIVIN study | 2005 | - | 1242 | 15-49 | Measured | National |
|  | DHS | 2007 | - | 19141 | 15-49 | Measured | National |
|  |  |  |  |  |  |  |  |
|  |  |  |  |  |  |  |  |
| **Panama** |  |  |  |  |  |  |  |
|  | WHO; Diaz et al. | 1999 | 262 | 613 | 15-93 | Self-reported | National |
|  | Situatcion Nutricinal, Segunda Encuesta de Niveles de Vida 2003. | 2003 | 7121 | 7364 | 18-75+ | Measured | National |
| **Peru** |  |  |  |  |  |  |  |
|  | WHO BMI database & DHS survey | 1992 | - | 4631 | 15-49 | Measured | National |
|  | WHO BMI database & DHS survey | 1996 | - | 9389 | 15-49 | Measured | National |
|  | WHO BMI database & DHS survey | 2000 | - | 25319 | 15-49 | Measured | National |
|  | WHO BMI database & DHS survey | 2006 | - | 796 | 15-49 | Measured | National |
| **Uruguay** |  |  |  |  |  |  |  |
|  | ENSO I; Pisabarro et al, 2002 | 1998 | 508 | 392 | 18-100 | Measured | National |
