# Supplementary material for: High Rates of Obesity and Non-Communicable Diseases Predicted across Latin America
Source: PLoS One. 2012 Aug 13;7(8):e39589. doi: 10.1371/journal.pone.0039589 (PMC3418261; doi:10.1371/journal.pone.0039589)
Supplement: Table S2 — References used for each disease in each country. (DOCX) [file pone.0039589.s003.docx]

Table S2 References used for each disease in each country

| **Country** | **Disease** | **Incidence** |  | | **Mortality** | **Survival** |
| --- | --- | --- | --- | --- | --- | --- |
| **Argentina** |  |  | |  | |  |
|  | **Diabetes** | Mexico proxy | | non-fatal | | non-fatal |
|  | **CHD** | Mexico proxy | | WHO 2008 | | US proxy |
|  | **Stroke** | Chile proxy | | WHO 2008 | | US proxy |
|  | **Hypertension** | Mexico proxy | | non-fatal | | non-fatal |
|  | **Cancers** |  | |  | |  |
|  | **Breast** | Globocan 2008 | | Globocan 2008 | | Costa Rica proxy |
|  | **Colorectal** | Globocan 2008 | | Globocan 2008 | | Cuba proxy |
|  | **Endometrial** | Globocan 2008 | | Globocan 2008 | | Brazil proxy, Viana 2006 |
|  | **Kidney** | Globocan 2008 | | Globocan 2008 | | Cuba proxy |
|  | **Liver** | Globocan 2008 | | Globocan 2008 | | Puerto Rico |
|  | **Oesophagus** | Globocan 2008 | | Globocan 2008 | | SEER |
|  | **Pancreatic** | Globocan 2008 | | Globocan 2008 | | SEER |
|  | **Osteoarthritis** | US proxy | | non-fatal | | non-fatal |
| **Bolivia** |  |  | |  | |  |
|  | **Diabetes** | Mexico proxy | | non-fatal | | non-fatal |
|  | **CHD** | Mexico proxy | | WHO 2008 | | SEER |
|  | **Stroke** | Chile proxy | | WHO 2008 | | SEER |
|  | **Hypertension** | Mexico proxy | | non-fatal | | non-fatal |
|  | **Cancers** |  | |  | |  |
|  | **Breast** | Globocan 2008 | | Globocan 2008 | | Costa Rica proxy |
|  | **Colorectal** | Globocan 2008 | | Globocan 2008 | | Cuba proxy |
|  | **Endometrial** | Globocan 2008 | | Globocan 2008 | | Brazil proxy, Viana 2006 |
|  | **Kidney** | Globocan 2008 | | Globocan 2008 | | Cuba proxy |
|  | **Liver** | Globocan 2008 | | Globocan 2008 | | Puerto Rico |
|  | **Oesophagus** | Globocan 2008 | | Globocan 2008 | | SEER |
|  | **Pancreatic** | Globocan 2008 | | Globocan 2008 | | SEER |
|  | **Osteoarthritis** | US proxy | | non-fatal | | non-fatal |
| **Chile** |  |  | |  | |  |
|  | **Diabetes** | Mexico proxy | | non-fatal | | non-fatal |
|  | **CHD** | Mexico proxy | | WHO 2008 | | SEER |
|  | **Stroke** | Lavados et al, 2005 | | WHO 2008 | | SEER |
|  | **Hypertension** | Mexico proxy | | non-fatal | | non-fatal |
|  | **Cancers** |  | |  | |  |
|  | **Breast** | Globocan 2008 | | Globocan 2008 | | Costa Rica proxy |
|  | **Colorectal** | Globocan 2008 | | Globocan 2008 | | Cuba proxy |
|  | **Endometrial** | Globocan 2008 | | Globocan 2008 | | Brazil proxy, Viana 2006 |
|  | **Kidney** | Globocan 2008 | | Globocan 2008 | | Cuba proxy |
|  | **Liver** | Globocan 2008 | | Globocan 2008 | | Puerto Rico |
|  | **Oesophagus** | Globocan 2008 | | Globocan 2008 | | SEER |
|  | **Pancreatic** | Globocan 2008 | | Globocan 2008 | | SEER |
|  | **Osteoarthritis** | US proxy | | non-fatal | | non-fatal |
|  |  |  | |  | |  |
|  |  |  | |  | |  |
| **Colombia** |  |  | |  | |  |
|  | **Diabetes** | Mexico proxy | | non-fatal | | non-fatal |
|  | **CHD** | Mexico proxy | | WHO 2008 | | US proxy |
|  | **Stroke** | Chile proxy | | WHO 2008 | | US proxy |
|  | **Hypertension** | Mexico proxy | | non-fatal | | non-fatal |
|  | **Cancers** |  | |  | |  |
|  | **Breast** | Globocan 2008 | | Globocan 2008 | | Costa Rica proxy |
|  | **Colorectal** | Globocan 2008 | | Globocan 2008 | | Cuba proxy |
|  | **Endometrial** | Globocan 2008 | | Globocan 2008 | | Brazil proxy, Viana 2006 |
|  | **Kidney** | Globocan 2008 | | Globocan 2008 | | Cuba proxy |
|  | **Liver** | Globocan 2008 | | Globocan 2008 | | Puerto Rico |
|  | **Oesophagus** | Globocan 2008 | | Globocan 2008 | | US proxy |
|  | **Pancreatic** | Globocan 2008 | | Globocan 2008 | | US proxy |
|  | **Osteoarthritis** | US proxy | | non-fatal | | non-fatal |
| **Costa Rica** |  |  | |  | |  |
|  | **Diabetes** | Mexico proxy | | non-fatal | | non-fatal |
|  | **CHD** | Mexico proxy | | WHO 2008 | | US proxy |
|  | **Stroke** | Chile proxy | | WHO 2008 | | US proxy |
|  | **Hypertension** | Mexico proxy | | non-fatal | | non-fatal |
|  | **Cancers** |  | |  | |  |
|  | **Breast** | Globocan 2008 | | Globocan 2008 | | Ortiz-Barboza et al 2011 |
|  | **Colorectal** | Globocan 2008 | | Globocan 2008 | | Cuba proxy |
|  | **Endometrial** | Globocan 2008 | | Globocan 2008 | | Brazil proxy, Viana 2006 |
|  | **Kidney** | Globocan 2008 | | Globocan 2008 | | Cuba proxy |
|  | **Liver** | Globocan 2008 | | Globocan 2008 | | Puerto Rico |
|  | **Oesophagus** | Globocan 2008 | | Globocan 2008 | | US proxy |
|  | **Pancreatic** | Globocan 2008 | | Globocan 2008 | | US proxy |
|  | **Osteoarthritis** | US proxy | | non-fatal | | non-fatal |
| **Cuba** |  |  | |  | |  |
|  | **Diabetes** | Mexico proxy | | non-fatal | | non-fatal |
|  | **CHD** | Mexico proxy | | WHO 2008 | | US proxy |
|  | **Stroke** | Chile proxy | | WHO 2008 | | US proxy |
|  | **Hypertension** | Mexico proxy | | non-fatal | | non-fatal |
|  | **Cancers** |  | |  | |  |
|  | **Breast** | Globocan 2008 | | Globocan 2008 | | Ortiz-Barboza et al 2011 |
|  | **Colorectal** | Globocan 2008 | | Globocan 2008 | | Coleman et al, 2008 |
|  | **Endometrial** | Globocan 2008 | | Globocan 2008 | | Brazil proxy, Viana 2006 |
|  | **Kidney** | Globocan 2008 | | Globocan 2008 | | Arez Valdez 2009 |
|  | **Liver** | Globocan 2008 | | Globocan 2008 | | Puerto Rico |
|  | **Oesophagus** | Globocan 2008 | | Globocan 2008 | | US proxy |
|  | **Pancreatic** | Globocan 2008 | | Globocan 2008 | | US proxy |
|  | **Osteoarthritis** | US proxy | | non-fatal | | non-fatal |
|  |  |  | |  | |  |
|  |  |  | |  | |  |
|  |  |  | |  | |  |
| **Nicaragua** |  |  | |  | |  |
|  | **Diabetes** | Mexico proxy | | non-fatal | | non-fatal |
|  | **CHD** | Mexico proxy | | WHO 2008 | | US proxy |
|  | **Stroke** | Chile proxy | | WHO 2008 | | US proxy |
|  | **Hypertension** | Mexico proxy | | non-fatal | | non-fatal |
|  | **Cancers** |  | |  | |  |
|  | **Breast** | Globocan 2008 | | Globocan 2008 | | Costa Rica proxy |
|  | **Colorectal** | Globocan 2008 | | Globocan 2008 | | Cuba proxy |
|  | **Endometrial** | Globocan 2008 | | Globocan 2008 | | Brazil proxy, Viana 2006 |
|  | **Kidney** | Globocan 2008 | | Globocan 2008 | | Cuba proxy |
|  | **Liver** | Globocan 2008 | | Globocan 2008 | | Puerto Rico |
|  | **Oesophagus** | Globocan 2008 | | Globocan 2008 | | US proxy |
|  | **Pancreatic** | Globocan 2008 | | Globocan 2008 | | US proxy |
|  | **Osteoarthritis** | US proxy | | non-fatal | | non-fatal |
| **Panama** |  |  | |  | |  |
|  | **Diabetes** | Mexico proxy | | non-fatal | | non-fatal |
|  | **CHD** | Mexico proxy | | WHO 2008 | | US proxy |
|  | **Stroke** | Chile proxy | | WHO 2008 | | US proxy |
|  | **Hypertension** | Mexico proxy | | non-fatal | | non-fatal |
|  | **Cancers** |  | |  | |  |
|  | **Breast** | Globocan 2008 | | Globocan 2008 | | Costa Rica proxy |
|  | **Colorectal** | Globocan 2008 | | Globocan 2008 | | Cuba proxy |
|  | **Endometrial** | Globocan 2008 | | Globocan 2008 | | Brazil proxy, Viana 2006 |
|  | **Kidney** | Globocan 2008 | | Globocan 2008 | | Cuba proxy |
|  | **Liver** | Globocan 2008 | | Globocan 2008 | | Puerto Rico |
|  | **Oesophagus** | Globocan 2008 | | Globocan 2008 | | US proxy |
|  | **Pancreatic** | Globocan 2008 | | Globocan 2008 | | US proxy |
|  | **Osteoarthritis** | US proxy | | non-fatal | | non-fatal |
| **Peru** |  |  | |  | |  |
|  | **Diabetes** | Mexico proxy | | non-fatal | | non-fatal |
|  | **CHD** | Mexico proxy | | WHO 2008 | | US proxy |
|  | **Stroke** | Chile proxy | | WHO 2008 | | US proxy |
|  | **Hypertension** |  | | non-fatal | | non-fatal |
|  | **Cancers** |  | |  | |  |
|  | **Breast** | Globocan 2008 | | Globocan 2008 | | Costa Rica proxy |
|  | **Colorectal** | Globocan 2008 | | Globocan 2008 | | Cuba proxy |
|  | **Endometrial** | Globocan 2008 | | Globocan 2008 | | Brazil proxy, Viana 2006 |
|  | **Kidney** | Globocan 2008 | | Globocan 2008 | | Cuba proxy |
|  | **Liver** | Globocan 2008 | | Globocan 2008 | | Puerto Rico |
|  | **Oesophagus** | Globocan 2008 | | Globocan 2008 | | US proxy |
|  | **Pancreatic** | Globocan 2008 | | Globocan 2008 | | US proxy |
|  | **Osteoarthritis** | US proxy | | non-fatal | | non-fatal |
|  |  |  | |  | |  |
|  |  |  | |  | |  |
|  |  |  | |  | |  |
| **Uruguay** |  |  | |  | |  |
|  | **Diabetes** | Mexico proxy | | non-fatal | | non-fatal |
|  | **CHD** | Mexico proxy | | WHO 2008 | | US proxy |
|  | **Stroke** | Chile proxy | | WHO 2008 | | US proxy |
|  | **Hypertension** | Mexico proxy | | non-fatal | | non-fatal |
|  | **Cancers** |  | |  | |  |
|  | **Breast** | Globocan 2008 | | Globocan 2008 | | Costa Rica proxy |
|  | **Colorectal** | Globocan 2008 | | Globocan 2008 | | Cuba proxy |
|  | **Endometrial** | Globocan 2008 | | Globocan 2008 | | Brazil proxy, Viana 2006 |
|  | **Kidney** | Globocan 2008 | | Globocan 2008 | | Cuba proxy |
|  | **Liver** | Globocan 2008 | | Globocan 2008 | | Puerto Rico |
|  | **Oesophagus** | Globocan 2008 | | Globocan 2008 | | US proxy |
|  | **Pancreatic** | Globocan 2008 | | Globocan 2008 | | US proxy |
|  | **Osteoarthritis** | US proxy | | non-fatal | | non-fatal |
