# Supplementary material for: High Rates of Obesity and Non-Communicable Diseases Predicted across Latin America
Source: PLoS One. 2012 Aug 13;7(8):e39589. doi: 10.1371/journal.pone.0039589 (PMC3418261; doi:10.1371/journal.pone.0039589)
Supplement: Table S3 — Incidence, mortality and survival input data for each country. (DOCX) [file pone.0039589.s004.docx]

Table S3 Incidence, mortality and survival input data for each country

Argentina incidence per 100,000

| breast cancer | |  |  |  |  |  |  |  |  |  |
| --- | --- | --- | --- | --- | --- | --- | --- | --- | --- | --- |
| AgeGp | 0-14 | 15-39 | 40-44 | 45-49 | 50-54 | 55-59 | 60-64 | 65-69 | 70-74 | >74 |
| M | 0 | 0 | 0 | 0 | 0 | 0 | 0 | 0 | 0 | 0 |
| F | 0 | 16.5 | 112.6 | 176.8 | 230.2 | 247.2 | 246.1 | 244.5 | 276.5 | 322 |
| colorectal cancer | |  |  |  |  |  |  |  |  |  |
| AgeGp | 0-14 | 15-39 | 40-44 | 45-49 | 50-54 | 55-59 | 60-64 | 65-69 | 70-74 | >74 |
| M | 0 | 2.1 | 10.4 | 20.8 | 38.9 | 62.8 | 99.7 | 146.4 | 205.8 | 286.4 |
| F | 0.1 | 1.7 | 12.6 | 20.7 | 31.6 | 43.2 | 62 | 78.2 | 112.4 | 184.4 |
| coronary heart disease | | |  |  |  |  |  |  |  |  |
| AgeGp | 0-19 | 20-24 | 25-44 | 45-49 | 50-59 | 60-64 | >64 |  |  |  |
| M | 0 | 4.66 | 21.01 | 74.64 | 169.17 | 321.73 | 418.75 |  |  |  |
| F | 0 | 3.75 | 13.61 | 46.75 | 105.02 | 198.04 | 263.02 |  |  |  |
| diabetes |  |  |  |  |  |  |  |  |  |  |
| AgeGp | 0-19 | 20-24 | 25-44 | 45-49 | 50-59 | 60-64 | >64 |  |  |  |
| M | 0 | 23.24 | 236.7 | 754.72 | 4384.45 | 8109.97 | 1043.3 |  |  |  |
| F | 0 | 31 | 241.37 | 624.23 | 365.16 | 516.26 | 853.98 |  |  |  |
| endometrial cancer | | |  |  |  |  |  |  |  |  |
| AgeGp | 0-14 | 15-39 | 40-44 | 45-49 | 50-54 | 55-59 | 60-64 | 65-69 | 70-74 | >74 |
| M | 0 | 0 | 0 | 0 | 0 | 0 | 0 | 0 | 0 | 0 |
| F | 0 | 0.6 | 4.4 | 5.7 | 11 | 14.1 | 22.1 | 26.4 | 34.4 | 37.1 |
| hypertension | |  |  |  |  |  |  |  |  |  |
| AgeGp | 0-19 | 20-24 | 25-44 | 45-49 | 50-59 | 60-64 | >64 |  |  |  |
| M | 0 | 38.64 | 273.17 | 736.86 | 2516.45 | 4739.1 | 1596.36 |  |  |  |
| F | 0 | 35.18 | 325.15 | 927.41 | 752.48 | 1012.9 | 1771.83 |  |  |  |
| kidney cancer | |  |  |  |  |  |  |  |  |  |
| AgeGp | 0-14 | 15-39 | 40-44 | 45-49 | 50-54 | 55-59 | 60-64 | 65-69 | 70-74 | >74 |
| M | 0.8 | 0.5 | 5.2 | 11.4 | 18 | 29.7 | 42.8 | 54.4 | 61.8 | 64.1 |
| F | 0.5 | 0.3 | 2.4 | 4.7 | 6.7 | 9.3 | 14.4 | 17.4 | 20.2 | 24.5 |
| liver cancer | |  |  |  |  |  |  |  |  |  |
| AgeGp | 0-14 | 15-39 | 40-44 | 45-49 | 50-54 | 55-59 | 60-64 | 65-69 | 70-74 | >74 |
| M | 0.1 | 0.2 | 1.4 | 2.8 | 5.4 | 10.7 | 18.4 | 27.7 | 39.7 | 55.6 |
| F | 0.1 | 0.3 | 0.9 | 2 | 3.9 | 6.1 | 8.9 | 13.5 | 18.1 | 30.5 |
| oesophageal cancer | | |  |  |  |  |  |  |  |  |
| AgeGp | 0-14 | 15-39 | 40-44 | 45-49 | 50-54 | 55-59 | 60-64 | 65-69 | 70-74 | >74 |
| M | 0 | 0.1 | 2.5 | 5.5 | 11.8 | 18.2 | 29.7 | 39.7 | 55.1 | 78 |
| F | 0 | 0.1 | 1.4 | 2.2 | 3.1 | 5.6 | 8.3 | 11.9 | 16.6 | 36.4 |
| osteoarthritis | |  |  |  |  |  |  |  |  |  |
| AgeGp | 0-9 | Oct-19 | 20-29 | 30-39 | 40-49 | 50-59 | 60-69 | 70-79 | 80-89 | >89 |
| M | 55.79 | 148.37 | 387.67 | 681.66 | 1026.52 | 1621.18 | 1782.75 | 1747.42 | 1696.52 | 1206.01 |
| F | 45.09 | 187.01 | 363.99 | 761.67 | 1365.24 | 2048.02 | 1976.92 | 1725.93 | 1691.07 | 848.47 |
| pancreatic cancer | |  |  |  |  |  |  |  |  |  |
| AgeGp | 0-14 | 15-39 | 40-44 | 45-49 | 50-54 | 55-59 | 60-64 | 65-69 | 70-74 | >74 |
| M | 0 | 0.3 | 2.5 | 6.3 | 12.6 | 21.4 | 35.6 | 50.6 | 68.2 | 103 |
| F | 0 | 0.2 | 2.1 | 4.4 | 8.7 | 15.4 | 23.4 | 34 | 52.1 | 87.6 |
| stroke |  |  |  |  |  |  |  |  |  |  |
| AgeGp | 0-24 | 25-34 | 35-44 | 45-54 | 55-64 | 65-74 | 75-84 | >84 |  |  |
| M | 7.44 | 8.57 | 31.01 | 118.46 | 411.85 | 485.2 | 1118.08 | 1557.65 |  |  |
| F | 1.08 | 2.93 | 12.18 | 71.75 | 182.45 | 407.57 | 910.33 | 994 |  |  |

Argentina mortality per 100,000

| breast cancer | |  |  |  |  |  |  |  |  |  |
| --- | --- | --- | --- | --- | --- | --- | --- | --- | --- | --- |
| AgeGp | 0-14 | 15-39 | 40-44 | 45-49 | 50-54 | 55-59 | 60-64 | 65-69 | 70-74 | >74 |
| M | 0 | 0 | 0 | 0 | 0 | 0 | 0 | 0 | 0 | 0 |
| F | 0 | 2.5 | 19.5 | 32 | 49.2 | 63.3 | 74.1 | 83.2 | 103.2 | 176.2 |
| colorectal cancer | |  |  |  |  |  |  |  |  |  |
| AgeGp | 0-14 | 15-39 | 40-44 | 45-49 | 50-54 | 55-59 | 60-64 | 65-69 | 70-74 | >74 |
| M | 0 | 0.8 | 4.4 | 8.8 | 17.8 | 31.1 | 53 | 84.2 | 130.9 | 234.9 |
| F | 0 | 0.8 | 4.3 | 8.3 | 13.7 | 20.3 | 31.2 | 42.5 | 67.6 | 147.7 |
| coronary heart disease | | |  |  |  |  |  |  |  |  |
| AgeGp | 0-14 | 15-29 | 30-44 | 45-59 | 60-69 | 70-79 | >79 |  |  |  |
| M | 0.11 | 7.17 | 21.5 | 71.68 | 184.27 | 414.62 | 1499.91 |  |  |  |
| F | 0.02 | 1.75 | 5.24 | 17.48 | 91.75 | 246.65 | 965.16 |  |  |  |
| diabetes |  |  |  |  |  |  |  |  |  |  |
| non terminal | |  |  |  |  |  |  |  |  |  |
| endometrial cancer | |  |  |  |  |  |  |  |  |  |
| AgeGp | 0-14 | 15-39 | 40-44 | 45-49 | 50-54 | 55-59 | 60-64 | 65-69 | 70-74 | >74 |
| M | 0 | 0 | 0 | 0 | 0 | 0 | 0 | 0 | 0 | 0 |
| F | 0 | 0.1 | 0.6 | 1.2 | 3 | 4.8 | 8.8 | 11.8 | 17.1 | 27.3 |
| hypertension | |  |  |  |  |  |  |  |  |  |
| non terminal | |  |  |  |  |  |  |  |  |  |
| kidney cancer | |  |  |  |  |  |  |  |  |  |
| AgeGp | 0-14 | 15-39 | 40-44 | 45-49 | 50-54 | 55-59 | 60-64 | 65-69 | 70-74 | >74 |
| M | 0.1 | 0.2 | 2 | 4.8 | 8.8 | 15.3 | 23.4 | 31.9 | 40.2 | 49.6 |
| F | 0.1 | 0.1 | 0.7 | 2 | 3.2 | 4.8 | 7.9 | 10.2 | 13.1 | 19.2 |
| liver cancer | |  |  |  |  |  |  |  |  |  |
| AgeGp | 0-14 | 15-39 | 40-44 | 45-49 | 50-54 | 55-59 | 60-64 | 65-69 | 70-74 | >74 |
| M | 0.1 | 0.2 | 1.2 | 2.6 | 5.2 | 10.2 | 17.6 | 26.5 | 38.6 | 54.6 |
| F | 0.1 | 0.2 | 1.1 | 1.9 | 3.7 | 5.9 | 8.6 | 12.9 | 17.6 | 30.1 |
| oesophageal cancer | |  |  |  |  |  |  |  |  |  |
| AgeGp | 0-14 | 15-39 | 40-44 | 45-49 | 50-54 | 55-59 | 60-64 | 65-69 | 70-74 | >74 |
| M | 0 | 0.1 | 1.4 | 4.2 | 9.3 | 14.7 | 25.1 | 35.3 | 50.1 | 72.1 |
| F | 0 | 0.1 | 0.7 | 1.4 | 2.1 | 3.9 | 6 | 9.1 | 12.9 | 29.1 |
| osteoarthritis | |  |  |  |  |  |  |  |  |  |
| non terminal | |  |  |  |  |  |  |  |  |  |
| pancreatic cancer | |  |  |  |  |  |  |  |  |  |
| AgeGp | 0-14 | 15-39 | 40-44 | 45-49 | 50-54 | 55-59 | 60-64 | 65-69 | 70-74 | >74 |
| M | 0 | 0.3 | 2.3 | 5.8 | 11.7 | 20.2 | 34.1 | 49.5 | 67.3 | 101.8 |
| F | 0 | 0.2 | 1.7 | 4 | 7.9 | 14.2 | 22.1 | 32.6 | 50.5 | 84.9 |
| stroke |  |  |  |  |  |  |  |  |  |  |
| AgeGp | 0-14 | 15-29 | 30-44 | 45-59 | 60-69 | 70-79 | >79 |  |  |  |
| M | 0.75 | 5.33 | 14.47 | 30.45 | 130.39 | 262.13 | 699.02 |  |  |  |
| F | 0.4 | 4.03 | 8.06 | 20.15 | 109.18 | 207.16 | 643.88 |  |  |  |

Bolivia incidence per 100,000

| breast cancer | |  |  |  |  |  |  |  |  |  |
| --- | --- | --- | --- | --- | --- | --- | --- | --- | --- | --- |
| AgeGp | 0-14 | 15-39 | 40-44 | 45-49 | 50-54 | 55-59 | 60-64 | 65-69 | 70-74 | >74 |
| F | 0 | 5.4 | 42.1 | 55 | 70.9 | 76.1 | 77.6 | 80.2 | 93.4 | 112.4 |
| colorectal cancer | |  |  |  |  |  |  |  |  |  |
| AgeGp | 0-14 | 15-39 | 40-44 | 45-49 | 50-54 | 55-59 | 60-64 | 65-69 | 70-74 | >74 |
| F | 0 | 1.5 | 3.6 | 4.7 | 15.1 | 13.5 | 17.7 | 54.2 | 37.6 | 55.1 |
| coronary heart disease | | |  |  |  |  |  |  |  |  |
| AgeGp | 0-19 | 20-24 | 25-44 | 45-49 | 50-59 | 60-64 | >64 |  |  |  |
| F | 0 | 6.07 | 22.04 | 75.67 | 170.01 | 320.6 | 425.79 |  |  |  |
| diabetes |  |  |  |  |  |  |  |  |  |  |
| AgeGp | 0-19 | 20-24 | 25-44 | 45-49 | 50-59 | 60-64 | >64 |  |  |  |
| F | 0 | 32.68 | 239.83 | 1100.84 | 645.3 | 796.1 | 1300.88 |  |  |  |
| endometrial cancer | |  |  |  |  |  |  |  |  |  |
| AgeGp | 0-14 | 15-39 | 40-44 | 45-49 | 50-54 | 55-59 | 60-64 | 65-69 | 70-74 | >74 |
| F | 0 | 0.5 | 6.9 | 12.3 | 8.4 | 14.1 | 11.8 | 7.6 | 13.9 | 5.7 |
| hypertension | |  |  |  |  |  |  |  |  |  |
| AgeGp | 0-19 | 20-24 | 25-44 | 45-49 | 50-59 | 60-64 | >64 |  |  |  |
| F | 0 | 37.65 | 358.47 | 1403.06 | 1130.1 | 1414.16 | 2473.74 |  |  |  |
| kidney cancer | |  |  |  |  |  |  |  |  |  |
| AgeGp | 0-14 | 15-39 | 40-44 | 45-49 | 50-54 | 55-59 | 60-64 | 65-69 | 70-74 | >74 |
| F | 0.2 | 0.1 | 0.4 | 0.9 | 3.9 | 2.7 | 2.5 | 6.5 | 11.2 | 6.9 |
| liver cancer | |  |  |  |  |  |  |  |  |  |
| AgeGp | 0-14 | 15-39 | 40-44 | 45-49 | 50-54 | 55-59 | 60-64 | 65-69 | 70-74 | >74 |
| F | 0.2 | 0.7 | 1.2 | 5.2 | 7.8 | 20.2 | 19.4 | 24.9 | 33.5 | 43.6 |
| oesophageal cancer | | |  |  |  |  |  |  |  |  |
| AgeGp | 0-14 | 15-39 | 40-44 | 45-49 | 50-54 | 55-59 | 60-64 | 65-69 | 70-74 | >74 |
| F | 0 | 0.1 | 0 | 1.9 | 0.6 | 0.7 | 1.7 | 5.4 | 8.4 | 4.6 |
| osteoarthritis | |  |  |  |  |  |  |  |  |  |
| AgeGp | 0-9 | Oct-19 | 20-29 | 30-39 | 40-49 | 50-59 | 60-69 | 70-79 | 80-89 | >89 |
| F | 45.09 | 180.77 | 380.01 | 877.53 | 1758.44 | 2727.98 | 2521.88 | 2201.71 | 2157.24 | 1082.36 |
| pancreatic cancer | |  |  |  |  |  |  |  |  |  |
| AgeGp | 0-14 | 15-39 | 40-44 | 45-49 | 50-54 | 55-59 | 60-64 | 65-69 | 70-74 | >74 |
| F | 0 | 0.1 | 0.8 | 1.4 | 2.8 | 10.8 | 5.9 | 22.8 | 26.5 | 21.8 |
| stroke |  |  |  |  |  |  |  |  |  |  |
| AgeGp | 0-24 | 25-34 | 35-44 | 45-54 | 55-64 | 65-74 | 75-84 | >84 |  |  |
| F | 2.03 | 5.52 | 22.96 | 135.28 | 344 | 768.44 | 1716.34 | 1874.11 |  |  |

Bolivia mortality per 100,000

| breast cancer | |  |  |  |  |  |  |  |  |  |
| --- | --- | --- | --- | --- | --- | --- | --- | --- | --- | --- |
| AgeGp | 0-14 | 15-39 | 40-44 | 45-49 | 50-54 | 55-59 | 60-64 | 65-69 | 70-74 | >74 |
| F | 0 | 1.3 | 10.1 | 14.7 | 21.2 | 24.9 | 27 | 29.3 | 34.9 | 45.9 |
| colorectal cancer | |  |  |  |  |  |  |  |  |  |
| AgeGp | 0-14 | 15-39 | 40-44 | 45-49 | 50-54 | 55-59 | 60-64 | 65-69 | 70-74 | >74 |
| F | 0 | 0.6 | 1.6 | 1.9 | 6.7 | 6.7 | 9.3 | 30.4 | 23.7 | 40.1 |
| coronary heart disease | | |  |  |  |  |  |  |  |  |
| AgeGp | 0-14 | 15-29 | 30-44 | 45-59 | 60-69 | 70-79 | >79 |  |  |  |
| F | 0 | 2.85 | 6.41 | 16.37 | 150.64 | 407.07 | 1004.24 |  |  |  |
| diabetes |  |  |  |  |  |  |  |  |  |  |
| non terminal | |  |  |  |  |  |  |  |  |  |
| endometrial cancer | | |  |  |  |  |  |  |  |  |
| AgeGp | 0-14 | 15-39 | 40-44 | 45-49 | 50-54 | 55-59 | 60-64 | 65-69 | 70-74 | >74 |
| F | 0 | 0.1 | 0.4 | 1.4 | 1.1 | 3.4 | 4.2 | 3.3 | 7 | 3.4 |
| hypertension | |  |  |  |  |  |  |  |  |  |
| non terminal | |  |  |  |  |  |  |  |  |  |
| kidney cancer | |  |  |  |  |  |  |  |  |  |
| AgeGp | 0-14 | 15-39 | 40-44 | 45-49 | 50-54 | 55-59 | 60-64 | 65-69 | 70-74 | >74 |
| F | 0.1 | 0.1 | 0 | 0.5 | 2.2 | 1.3 | 1.7 | 4.3 | 8.4 | 5.7 |
| liver cancer | |  |  |  |  |  |  |  |  |  |
| AgeGp | 0-14 | 15-39 | 40-44 | 45-49 | 50-54 | 55-59 | 60-64 | 65-69 | 70-74 | >74 |
| F | 0.1 | 0.6 | 2 | 5.2 | 7.8 | 20.2 | 19.4 | 26 | 34.9 | 45.9 |
| oesophageal cancer | | |  |  |  |  |  |  |  |  |
| AgeGp | 0-39 | 40-44 | 45-49 | 50-54 | 55-59 | 60-64 | 65-69 | 70-74 | >74 |  |
| F | 0 | 0 | 1.4 | 0.6 | 0.7 | 1.7 | 4.3 | 7 | 3.4 |  |
| osteoarthritis | |  |  |  |  |  |  |  |  |  |
| non terminal | |  |  |  |  |  |  |  |  |  |
| pancreatic cancer | |  |  |  |  |  |  |  |  |  |
| AgeGp | 0-14 | 15-39 | 40-44 | 45-49 | 50-54 | 55-59 | 60-64 | 65-69 | 70-74 | >74 |
| F | 0 | 0.1 | 0.4 | 1.4 | 2.8 | 10.8 | 5.9 | 22.8 | 26.5 | 21.8 |
| stroke |  |  |  |  |  |  |  |  |  |  |
| AgeGp | 0-14 | 15-29 | 30-44 | 45-59 | 60-69 | 70-79 | >79 |  |  |  |
| F | 0.63 | 2.43 | 10.34 | 24.32 | 148.91 | 321.65 | 923.27 |  |  |  |

Chile incidence per 100,000

| breast cancer | |  |  |  |  |  |  |  |  |  |
| --- | --- | --- | --- | --- | --- | --- | --- | --- | --- | --- |
| AgeGp | 0-14 | 15-39 | 40-44 | 45-49 | 50-54 | 55-59 | 60-64 | 65-69 | 70-74 | >74 |
| M | 0 | 0 | 0 | 0 | 0 | 0 | 0 | 0 | 0 | 0 |
| F | 0 | 10.4 | 55.7 | 96.9 | 119.7 | 124.9 | 129.1 | 136.7 | 153.6 | 190.4 |
| colorectal cancer | |  |  |  |  |  |  |  |  |  |
| AgeGp | 0-14 | 15-39 | 40-44 | 45-49 | 50-54 | 55-59 | 60-64 | 65-69 | 70-74 | >74 |
| M | 0.1 | 1.3 | 5.3 | 11.4 | 15.7 | 28.6 | 54.1 | 88.4 | 123.2 | 183.2 |
| F | 0.1 | 1.2 | 7.2 | 10.2 | 17.6 | 24.7 | 43 | 65.7 | 80.7 | 159.3 |
| coronary heart disease | | |  |  |  |  |  |  |  |  |
| AgeGp | 0-19 | 20-24 | 25-44 | 45-49 | 50-59 | 60-64 | >64 |  |  |  |
| M | 0 | 3.3 | 14.9 | 52.93 | 119.97 | 228.16 | 296.97 |  |  |  |
| F | 0 | 2.87 | 10.41 | 35.74 | 80.3 | 151.41 | 201.1 |  |  |  |
| diabetes |  |  |  |  |  |  |  |  |  |  |
| AgeGp | 0-19 | 20-24 | 25-44 | 45-49 | 50-59 | 60-64 | >64 |  |  |  |
| M | 0 | 22.08 | 207.15 | 694.86 | 4084.89 | 8140.66 | 1044.61 |  |  |  |
| F | 0 | 32.65 | 274.9 | 1323.71 | 832.34 | 1015.2 | 1385.4 |  |  |  |
| endometrial cancer | | |  |  |  |  |  |  |  |  |
| AgeGp | 0-14 | 15-39 | 40-44 | 45-49 | 50-54 | 55-59 | 60-64 | 65-69 | 70-74 | >74 |
| M | 0 | 0 | 0 | 0 | 0 | 0 | 0 | 0 | 0 | 0 |
| F | 0 | 0.2 | 1 | 2.5 | 4.4 | 6.5 | 12 | 13.5 | 19.4 | 15.7 |
| hypertension | |  |  |  |  |  |  |  |  |  |
| AgeGp | 0-19 | 20-24 | 25-44 | 45-49 | 50-59 | 60-64 | >64 |  |  |  |
| M | 0 | 35.45 | 271.21 | 777.26 | 2662.89 | 5169.53 | 1717.42 |  |  |  |
| F | 0 | 38.42 | 385.48 | 1555.63 | 1308.76 | 1637.72 | 2586.89 |  |  |  |
| kidney cancer | |  |  |  |  |  |  |  |  |  |
| AgeGp | 0-14 | 15-39 | 40-44 | 45-49 | 50-54 | 55-59 | 60-64 | 65-69 | 70-74 | >74 |
| M | 0.6 | 0.4 | 2.9 | 6.4 | 13.6 | 21.6 | 32.2 | 48.6 | 61.6 | 69 |
| F | 0.5 | 0.3 | 1.6 | 2.8 | 7 | 8.8 | 15 | 21 | 24.8 | 34.8 |
| liver cancer | |  |  |  |  |  |  |  |  |  |
| AgeGp | 0-14 | 15-39 | 40-44 | 45-49 | 50-54 | 55-59 | 60-64 | 65-69 | 70-74 | >74 |
| M | 0.1 | 0.2 | 1 | 2.7 | 5.2 | 13.1 | 23.2 | 36.8 | 61.6 | 80.2 |
| F | 0.1 | 0.3 | 0.8 | 2.5 | 4 | 6.8 | 11.4 | 19.5 | 29.7 | 46.5 |
| oesophageal cancer | | |  |  |  |  |  |  |  |  |
| AgeGp | 0-39 | 40-44 | 45-49 | 50-54 | 55-59 | 60-64 | 65-69 | 70-74 | >74 |  |
| M | 0 | 0.6 | 1.5 | 4.3 | 8.3 | 18.6 | 33.3 | 54.8 | 95.7 |  |
| F | 0 | 0.6 | 1 | 2.2 | 4.8 | 9 | 18.8 | 31.1 | 67.7 |  |
| osteoarthritis | |  |  |  |  |  |  |  |  |  |
| AgeGp | 0-9 | Oct-19 | 20-29 | 30-39 | 40-49 | 50-59 | 60-69 | 70-79 | 80-89 | >89 |
| M | 55.79 | 148.37 | 372.11 | 758.54 | 1293.65 | 2026.69 | 2202.99 | 2126.46 | 2064.51 | 1467.61 |
| F | 45.09 | 187.01 | 405.3 | 897.69 | 1760.11 | 2942.13 | 2667.54 | 2260.36 | 2214.71 | 1111.19 |
| pancreatic cancer | |  |  |  |  |  |  |  |  |  |
| AgeGp | 0-14 | 15-39 | 40-44 | 45-49 | 50-54 | 55-59 | 60-64 | 65-69 | 70-74 | >74 |
| M | 0 | 0.3 | 1.4 | 3.5 | 7 | 14.1 | 21.6 | 32.4 | 45.6 | 63.8 |
| F | 0 | 0.1 | 0.8 | 2.8 | 8.4 | 13.1 | 28 | 29.3 | 42.8 | 67.7 |
| stroke |  |  |  |  |  |  |  |  |  |  |
| AgeGp | 0-24 | 25-34 | 35-44 | 45-54 | 55-64 | 65-74 | 75-84 | >84 |  |  |
| M | 7.64 | 8.8 | 31.85 | 121.68 | 423.05 | 498.39 | 1148.48 | 1600 |  |  |
| F | 1.14 | 3.11 | 12.94 | 76.25 | 193.9 | 433.13 | 967.41 | 1056.34 |  |  |

Chile mortality per 100,000

| breast cancer | |  |  |  |  |  |  |  |  |  |
| --- | --- | --- | --- | --- | --- | --- | --- | --- | --- | --- |
| AgeGp | 0-14 | 15-39 | 40-44 | 45-49 | 50-54 | 55-59 | 60-64 | 65-69 | 70-74 | >74 |
| M | 0 | 0 | 0 | 0 | 0 | 0 | 0 | 0 | 0 | 0 |
| F | 0 | 1.5 | 9.9 | 17.6 | 25.6 | 32 | 38.8 | 46.6 | 57.4 | 107 |
| colorectal cancer | |  |  |  |  |  |  |  |  |  |
| AgeGp | 0-14 | 15-39 | 40-44 | 45-49 | 50-54 | 55-59 | 60-64 | 65-69 | 70-74 | >74 |
| M | 0 | 0.6 | 2.1 | 4.9 | 7.2 | 14.1 | 28.9 | 50.8 | 78.2 | 150.9 |
| F | 0 | 0.6 | 2.4 | 4.1 | 7.6 | 11.6 | 21.7 | 35.7 | 48.6 | 127.5 |
| coronary heart disease | | |  |  |  |  |  |  |  |  |
| AgeGp | 0-14 | 15-29 | 30-44 | 45-59 | 60-69 | 70-79 | >79 |  |  |  |
| M | 0 | 4.13 | 16.5 | 41.25 | 127.99 | 336.25 | 1063 |  |  |  |
| F | 0 | 5.56 | 5.56 | 5.56 | 311.68 | 311.68 | 311.68 |  |  |  |
| diabetes |  |  |  |  |  |  |  |  |  |  |
| non terminal | |  |  |  |  |  |  |  |  |  |
| endometrial cancer | | |  |  |  |  |  |  |  |  |
| AgeGp | 0-39 | 40-44 | 45-49 | 50-54 | 55-59 | 60-64 | 65-69 | 70-74 | >74 |  |
| M | 0 | 0 | 0 | 0 | 0 | 0 | 0 | 0 | 0 |  |
| F | 0 | 0.2 | 0.5 | 1.2 | 2.3 | 4.8 | 6 | 9.7 | 11.4 |  |
| hypertension | |  |  |  |  |  |  |  |  |  |
| non terminal | |  |  |  |  |  |  |  |  |  |
| kidney cancer | |  |  |  |  |  |  |  |  |  |
| AgeGp | 0-39 | 40-44 | 45-49 | 50-54 | 55-59 | 60-64 | 65-69 | 70-74 | >74 |  |
| M | 0.1 | 1.1 | 2.7 | 6.6 | 11.2 | 17.6 | 28.5 | 40 | 53.5 |  |
| F | 0.1 | 0.6 | 1.1 | 3.4 | 4.5 | 8.1 | 12.4 | 16 | 27.1 |  |
| liver cancer | |  |  |  |  |  |  |  |  |  |
| AgeGp | 0-14 | 15-39 | 40-44 | 45-49 | 50-54 | 55-59 | 60-64 | 65-69 | 70-74 | >74 |
| M | 0.1 | 0.2 | 1 | 2.5 | 5 | 12.5 | 22.3 | 35.5 | 59.8 | 78.9 |
| F | 0.1 | 0.2 | 1 | 2.3 | 3.8 | 6.5 | 10.8 | 18.8 | 28.7 | 45.7 |
| oesophageal cancer | | |  |  |  |  |  |  |  |  |
| AgeGp | 0-39 | 40-44 | 45-49 | 50-54 | 55-59 | 60-64 | 65-69 | 70-74 | >74 |  |
| M | 0 | 0.5 | 1.2 | 3.3 | 6.7 | 15.6 | 29.8 | 49.9 | 88.8 |  |
| F | 0 | 0.3 | 0.7 | 1.4 | 3.3 | 6.6 | 14.3 | 24.3 | 54.4 |  |
| osteoarthritis | |  |  |  |  |  |  |  |  |  |
| non terminal | |  |  |  |  |  |  |  |  |  |
| pancreatic cancer | |  |  |  |  |  |  |  |  |  |
| AgeGp | 0-14 | 15-39 | 40-44 | 45-49 | 50-54 | 55-59 | 60-64 | 65-69 | 70-74 | >74 |
| M | 0 | 0.2 | 1.4 | 3.2 | 6.6 | 13.3 | 20.6 | 31.5 | 45 | 62.9 |
| F | 0 | 0 | 1.1 | 2.5 | 7.6 | 12.1 | 26.5 | 28.2 | 41.3 | 65.6 |
| stroke |  |  |  |  |  |  |  |  |  |  |
| AgeGp | 0-14 | 15-29 | 30-44 | 45-59 | 60-69 | 70-79 | >79 |  |  |  |
| M | 0.52 | 3.77 | 6.28 | 23.23 | 122.05 | 344.46 | 735.03 |  |  |  |
| F | 0.19 | 3.42 | 6.83 | 15.71 | 92.17 | 292.94 | 625.01 |  |  |  |

Colombia incidence per 100,000

| breast cancer | |  |  |  |  |  |  |  |  |  |
| --- | --- | --- | --- | --- | --- | --- | --- | --- | --- | --- |
| AgeGp | 0-14 | 15-39 | 40-44 | 45-49 | 50-54 | 55-59 | 60-64 | 65-69 | 70-74 | >74 |
| F | 0 | 7.1 | 44 | 62.3 | 79.6 | 91.8 | 101.3 | 130.5 | 168.2 | 161.5 |
| colorectal cancer | |  |  |  |  |  |  |  |  |  |
| AgeGp | 0-14 | 15-39 | 40-44 | 45-49 | 50-54 | 55-59 | 60-64 | 65-69 | 70-74 | >74 |
| F | 0 | 2 | 6 | 10.4 | 17.3 | 24.3 | 35.4 | 56.9 | 72.8 | 127.5 |
| coronary heart disease | | |  |  |  |  |  |  |  |  |
| AgeGp | 0-19 | 20-24 | 25-44 | 45-49 | 50-59 | 60-64 | >64 |  |  |  |
| F | 0 | 5.82 | 21.12 | 72.52 | 162.93 | 307.24 | 408.04 |  |  |  |
| diabetes |  |  |  |  |  |  |  |  |  |  |
| AgeGp | 0-19 | 20-24 | 25-44 | 45-49 | 50-59 | 60-64 | >64 |  |  |  |
| F | 0 | 30.47 | 188.78 | 930.74 | 563.46 | 701.03 | 1154 |  |  |  |
| endometrial cancer | | |  |  |  |  |  |  |  |  |
| AgeGp | 0-14 | 15-39 | 40-44 | 45-49 | 50-54 | 55-59 | 60-64 | 65-69 | 70-74 | >74 |
| F | 0 | 0.5 | 3.3 | 2.4 | 6.2 | 8.4 | 20.6 | 14.5 | 14.2 | 18.9 |
| hypertension | |  |  |  |  |  |  |  |  |  |
| AgeGp | 0-19 | 20-24 | 25-44 | 45-49 | 50-59 | 60-64 | >64 |  |  |  |
| F | 0 | 33.25 | 318.48 | 1293.3 | 1061.07 | 1327.77 | 2322.63 |  |  |  |
| kidney cancer | |  |  |  |  |  |  |  |  |  |
| AgeGp | 0-14 | 15-39 | 40-44 | 45-49 | 50-54 | 55-59 | 60-64 | 65-69 | 70-74 | >74 |
| F | 0.7 | 0.3 | 1.2 | 1.3 | 2.9 | 4.1 | 5.6 | 13.3 | 9.6 | 13.1 |
| liver cancer | |  |  |  |  |  |  |  |  |  |
| AgeGp | 0-14 | 15-39 | 40-44 | 45-49 | 50-54 | 55-59 | 60-64 | 65-69 | 70-74 | >74 |
| F | 0 | 0.3 | 0.4 | 1.5 | 2.6 | 4.9 | 8.7 | 15 | 19 | 30.3 |
| oesophageal cancer | | |  |  |  |  |  |  |  |  |
| AgeGp | 0-14 | 15-39 | 40-44 | 45-49 | 50-54 | 55-59 | 60-64 | 65-69 | 70-74 | >74 |
| F | 0 | 0.1 | 0.2 | 0.7 | 1.2 | 1.6 | 4.3 | 6.4 | 10.5 | 24.8 |
| osteoarthritis | |  |  |  |  |  |  |  |  |  |
| AgeGp | 15-24 | 25-34 | 35-44 | 45-54 | 55-64 | 65-74 | >74 |  |  |  |
| F | 4.04 | 14.15 | 33.82 | 44.7 | 51.65 | 48.2 | 31.29 |  |  |  |
| pancreatic cancer | |  |  |  |  |  |  |  |  |  |
| AgeGp | 0-14 | 15-39 | 40-44 | 45-49 | 50-54 | 55-59 | 60-64 | 65-69 | 70-74 | >74 |
| F | 0 | 0.2 | 0.6 | 1.7 | 3.3 | 5.1 | 11.1 | 14.8 | 25.8 | 44 |
| stroke |  |  |  |  |  |  |  |  |  |  |
| AgeGp | 0-24 | 25-34 | 35-44 | 45-54 | 55-64 | 65-74 | 75-84 | >84 |  |  |
| F | 1.25 | 3.41 | 14.2 | 83.63 | 212.67 | 475.06 | 1061.07 | 1158.6 |  |  |

Colombia mortality per 100,000

| breast cancer | |  |  |  |  |  |  |  |  |  |
| --- | --- | --- | --- | --- | --- | --- | --- | --- | --- | --- |
| AgeGp | 0-14 | 15-39 | 40-44 | 45-49 | 50-54 | 55-59 | 60-64 | 65-69 | 70-74 | >74 |
| F | 0 | 1.6 | 10.9 | 16.6 | 25.4 | 32.7 | 35.7 | 42.1 | 53.5 | 69.2 |
| colorectal cancer | |  |  |  |  |  |  |  |  |  |
| AgeGp | 0-14 | 15-39 | 40-44 | 45-49 | 50-54 | 55-59 | 60-64 | 65-69 | 70-74 | >74 |
| F | 0 | 0.7 | 3 | 4.7 | 8.4 | 12.5 | 19.4 | 33.2 | 45 | 83.8 |
| coronary heart disease | | |  |  |  |  |  |  |  |  |
| AgeGp | 0-14 | 15-29 | 30-44 | 45-45 | 46-59 | 60-69 | 70-79 | >79 |  |  |
| F | 0.08 | 3.11 | 9.33 | 25.67 | 25.67 | 172.38 | 422.32 | 1017.01 |  |  |
| diabetes |  |  |  |  |  |  |  |  |  |  |
| non terminal | |  |  |  |  |  |  |  |  |  |
| endometrial cancer | | |  |  |  |  |  |  |  |  |
| AgeGp | 0-14 | 15-39 | 40-49 | 50-54 | 55-59 | 60-64 | 65-69 | 70-74 | >74 |  |
| F | 0 | 0.1 | 0.4 | 1.2 | 1.7 | 4.3 | 4.1 | 6.5 | 8.9 |  |
| hypertension | |  |  |  |  |  |  |  |  |  |
| non terminal | |  |  |  |  |  |  |  |  |  |
| kidney cancer | |  |  |  |  |  |  |  |  |  |
| AgeGp | 0-14 | 15-39 | 40-44 | 45-49 | 50-54 | 55-59 | 60-64 | 65-69 | 70-74 | >74 |
| F | 0.2 | 0.1 | 0.6 | 0.5 | 1.2 | 1.7 | 2.3 | 5.6 | 4.5 | 7.8 |
| liver cancer | |  |  |  |  |  |  |  |  |  |
| AgeGp | 0-14 | 15-39 | 40-44 | 45-49 | 50-54 | 55-59 | 60-64 | 65-69 | 70-74 | >74 |
| F | 0.1 | 0.4 | 1.5 | 2.6 | 4.7 | 9.2 | 16.9 | 30.2 | 39.4 | 65.5 |
| oesophageal cancer | | |  |  |  |  |  |  |  |  |
| AgeGp | 0-14 | 15-39 | 40-44 | 45-49 | 50-54 | 55-59 | 60-64 | 65-69 | 70-74 | >74 |
| F | 0 | 0.1 | 0.2 | 0.7 | 1.1 | 1.4 | 4 | 6 | 9.6 | 22.9 |
| osteoarthritis | |  |  |  |  |  |  |  |  |  |
| non terminal | |  |  |  |  |  |  |  |  |  |
| pancreatic cancer | |  |  |  |  |  |  |  |  |  |
| AgeGp | 0-14 | 15-39 | 40-44 | 45-49 | 50-54 | 55-59 | 60-64 | 65-69 | 70-74 | >74 |
| F | 0 | 0.2 | 0.8 | 1.6 | 3.3 | 5.4 | 12.4 | 17.3 | 30.3 | 45.7 |
| stroke |  |  |  |  |  |  |  |  |  |  |
| AgeGp | 0-14 | 15-29 | 30-44 | 45-59 | 60-69 | 70-79 | >79 |  |  |  |
| F | 0.56 | 3.54 | 8.49 | 22.64 | 107.85 | 207.4 | 580.71 |  |  |  |

Costa Rica incidence per 100,000

| breast cancer | |  |  |  |  |  |  |  |  |  |
| --- | --- | --- | --- | --- | --- | --- | --- | --- | --- | --- |
| AgeGp | 0-14 | 15-39 | 40-44 | 45-49 | 50-54 | 55-59 | 60-64 | 65-69 | 70-74 | >74 |
| M | 0 | 0 | 0 | 0 | 0 | 0 | 0 | 0 | 0 | 0 |
| F | 0 | 8.6 | 51.4 | 90.4 | 103 | 140.3 | 163.6 | 189.6 | 184.3 | 227.7 |
| colorectal cancer | |  |  |  |  |  |  |  |  |  |
| AgeGp | 0-14 | 15-39 | 40-44 | 45-49 | 50-54 | 55-59 | 60-64 | 65-69 | 70-74 | >74 |
| M | 0 | 2.2 | 5.6 | 9.6 | 20.7 | 33.9 | 59 | 93.5 | 122.2 | 200.4 |
| F | 0 | 1.8 | 7.8 | 14.3 | 19.5 | 29.2 | 46.1 | 58.3 | 85.5 | 188.7 |
| coronary heart disease | | |  |  |  |  |  |  |  |  |
| AgeGp | 0-19 | 20-24 | 25-44 | 45-49 | 50-59 | 60-64 | >64 |  |  |  |
| M | 0 | 4.18 | 18.86 | 67 | 151.85 | 288.77 | 375.87 |  |  |  |
| F | 0 | 4.72 | 17.12 | 58.81 | 132.13 | 249.15 | 330.9 |  |  |  |
| diabetes |  |  |  |  |  |  |  |  |  |  |
| AgeGp | 0-19 | 20-24 | 25-44 | 45-49 | 50-59 | 60-64 | >64 |  |  |  |
| M | 0 | 22.99 | 188.84 | 678.47 | 3914.25 | 7508.3 | 973.62 |  |  |  |
| F | 0 | 32.2 | 234.73 | 1026.97 | 650.4 | 853.78 | 1392.94 |  |  |  |
| endometrial cancer | | |  |  |  |  |  |  |  |  |
| AgeGp | 0-14 | 15-39 | 40-44 | 45-49 | 50-54 | 55-59 | 60-64 | 65-69 | 70-74 | >74 |
| M | 0 | 0 | 0 | 0 | 0 | 0 | 0 | 0 | 0 | 0 |
| F | 0 | 0.7 | 3.9 | 5.7 | 10.7 | 18.7 | 23.8 | 29.2 | 26.7 | 32.8 |
| hypertension | |  |  |  |  |  |  |  |  |  |
| AgeGp | 0-19 | 20-24 | 25-44 | 45-49 | 50-59 | 60-64 | >64 |  |  |  |
| M | 0 | 38.1 | 258.5 | 752.26 | 2540 | 4844.81 | 1631.96 |  |  |  |
| F | 0 | 37.71 | 352.03 | 1383.62 | 1161.72 | 1496.27 | 2617.39 |  |  |  |
| kidney cancer | |  |  |  |  |  |  |  |  |  |
| AgeGp | 0-14 | 15-39 | 40-44 | 45-49 | 50-54 | 55-59 | 60-64 | 65-69 | 70-74 | >74 |
| M | 0.5 | 0.6 | 1.2 | 3.4 | 5.2 | 8.2 | 11.5 | 15.6 | 17.9 | 21.4 |
| F | 0.7 | 0.2 | 2 | 2.9 | 2.7 | 4.7 | 6.4 | 10.4 | 10.7 | 9.4 |
| liver cancer | |  |  |  |  |  |  |  |  |  |
| AgeGp | 0-14 | 15-39 | 40-44 | 45-49 | 50-54 | 55-59 | 60-64 | 65-69 | 70-74 | >74 |
| M | 0.2 | 1 | 3.1 | 2.8 | 7.7 | 9.3 | 24.6 | 37.8 | 65.6 | 91.5 |
| F | 0.5 | 0.6 | 1.3 | 2.2 | 3.6 | 5.8 | 9.5 | 18.8 | 34.7 | 70.2 |
| oesophageal cancer | | |  |  |  |  |  |  |  |  |
| AgeGp | 0-44 | 45-49 | 50-54 | 55-59 | 60-64 | 65-69 | 70-74 | >74 |  |  |
| M | 0 | 0.7 | 0.9 | 3.5 | 4.9 | 8.9 | 20.9 | 25.3 |  |  |
| F | 0 | 0 | 0.9 | 2.3 | 0 | 2.1 | 5.3 | 9.4 |  |  |
| osteoarthritis | |  |  |  |  |  |  |  |  |  |
| AgeGp | 0-9 | Oct-19 | 20-29 | 30-39 | 40-49 | 50-59 | 60-69 | 70-79 | 80-89 | >89 |
| M | 55.79 | 148.37 | 379.27 | 725.25 | 1262.82 | 1890.21 | 2070.84 | 2029.81 | 1970.67 | 1400.9 |
| F | 45.09 | 187.01 | 385.49 | 843.27 | 1725.15 | 2811.36 | 2625.9 | 2292.53 | 2246.22 | 1127.01 |
| pancreatic cancer | |  |  |  |  |  |  |  |  |  |
| AgeGp | 0-14 | 15-39 | 40-44 | 45-49 | 50-54 | 55-59 | 60-64 | 65-69 | 70-74 | >74 |
| M | 0 | 0.2 | 3.1 | 3.4 | 7.7 | 10.5 | 19.7 | 26.7 | 44.7 | 83.7 |
| F | 0 | 0.2 | 2.6 | 3.6 | 3.6 | 7 | 12.7 | 20.8 | 32 | 92 |
| stroke |  |  |  |  |  |  |  |  |  |  |
| AgeGp | 0-24 | 25-34 | 35-44 | 45-54 | 55-64 | 65-74 | 75-84 | >84 |  |  |
| M | 4.11 | 4.73 | 17.14 | 65.47 | 227.62 | 268.16 | 617.93 | 860.87 |  |  |
| F | 0.87 | 2.36 | 9.84 | 57.99 | 147.47 | 329.43 | 735.79 | 803.42 |  |  |

Costa Rica mortality per 100,000

| breast cancer | |  |  |  |  |  |  |  |  |  |
| --- | --- | --- | --- | --- | --- | --- | --- | --- | --- | --- |
| AgeGp | 0-14 | 15-39 | 40-44 | 45-49 | 50-54 | 55-59 | 60-64 | 65-69 | 70-74 | >74 |
| M | 0 | 0 | 0 | 0 | 0 | 0 | 0 | 0 | 0 | 0 |
| F | 0 | 1.5 | 11.1 | 18.7 | 30.2 | 45.6 | 33.4 | 47.9 | 74.8 | 112.3 |
| colorectal cancer | |  |  |  |  |  |  |  |  |  |
| AgeGp | 0-14 | 15-39 | 40-44 | 45-49 | 50-54 | 55-59 | 60-64 | 65-69 | 70-74 | >74 |
| M | 0.2 | 1.3 | 5 | 9.6 | 10.3 | 16.4 | 24.6 | 49 | 62.6 | 144 |
| F | 0 | 0.7 | 3.9 | 5 | 10.7 | 17.5 | 27 | 35.4 | 50.7 | 135.7 |
| coronary heart disease | | |  |  |  |  |  |  |  |  |
| AgeGp | 0-14 | 15-29 | 30-44 | 45-59 | 60-69 | 70-79 | >79 |  |  |  |
| M | 0.18 | 2.01 | 4.69 | 12.06 | 70.28 | 144.77 | 463.82 |  |  |  |
| F | 0 | 2.32 | 5.8 | 14.49 | 110.45 | 276.13 | 1118.33 |  |  |  |
| diabetes |  |  |  |  |  |  |  |  |  |  |
| non terminal | |  |  |  |  |  |  |  |  |  |
| endometrial cancer | |  |  |  |  |  |  |  |  |  |
| AgeGp | 0-39 | 40-49 | 50-54 | 55-59 | 60-64 | 65-69 | 70-74 | >74 |  |  |
| M | 0 | 0 | 0 | 0 | 0 | 0 | 0 | 0 |  |  |
| F | 0 | 0.7 | 1.8 | 4.7 | 6.4 | 8.3 | 16 | 28.1 |  |  |
| hypertension | |  |  |  |  |  |  |  |  |  |
| non terminal | |  |  |  |  |  |  |  |  |  |
| kidney cancer | |  |  |  |  |  |  |  |  |  |
| AgeGp | 0-14 | 15-39 | 40-44 | 45-49 | 50-54 | 55-59 | 60-64 | 65-69 | 70-74 | >74 |
| M | 0 | 0.1 | 1.2 | 1.4 | 4.3 | 7 | 6.6 | 8.9 | 8.9 | 23.4 |
| F | 0.2 | 0.1 | 0 | 0.7 | 0.9 | 4.7 | 3.2 | 6.3 | 10.7 | 12.5 |
| liver cancer | |  |  |  |  |  |  |  |  |  |
| AgeGp | 0-14 | 15-39 | 40-44 | 45-49 | 50-54 | 55-59 | 60-64 | 65-69 | 70-74 | >74 |
| M | 0.2 | 0.8 | 1.9 | 2.8 | 6 | 12.9 | 19.7 | 37.8 | 53.6 | 95.4 |
| F | 0 | 0.3 | 0.7 | 2.2 | 1.8 | 7 | 15.9 | 20.8 | 45.4 | 68.6 |
| oesophageal cancer | |  |  |  |  |  |  |  |  |  |
| AgeGp | 0-44 | 45-49 | 50-54 | 55-59 | 60-64 | 65-69 | 70-74 | >74 |  |  |
| M | 0 | 1.4 | 1.7 | 5.8 | 8.2 | 13.4 | 20.9 | 38.9 |  |  |
| F | 0 | 0 | 0.9 | 1.2 | 1.6 | 2.1 | 2.7 | 15.6 |  |  |
| osteoarthritis | |  |  |  |  |  |  |  |  |  |
| non terminal | |  |  |  |  |  |  |  |  |  |
| pancreatic cancer | |  |  |  |  |  |  |  |  |  |
| AgeGp | 0-14 | 15-39 | 40-44 | 45-49 | 50-54 | 55-59 | 60-64 | 65-69 | 70-74 | >74 |
| M | 0 | 0.2 | 2.5 | 2.8 | 6.9 | 14 | 13.1 | 28.9 | 41.7 | 72 |
| F | 0 | 0.1 | 0 | 2.2 | 6.2 | 8.2 | 9.5 | 20.8 | 37.4 | 74.9 |
| stroke |  |  |  |  |  |  |  |  |  |  |
| AgeGp | 0-14 | 15-29 | 30-44 | 45-59 | 60-69 | 70-79 | >79 |  |  |  |
| M | 0.58 | 1.94 | 9.71 | 34.95 | 102.94 | 276.18 | 989.23 |  |  |  |
| F | 0.41 | 2.69 | 10.74 | 24.16 | 140.22 | 300.89 | 978.63 |  |  |  |

Cuba incidence per 100,000

| breast cancer | |  |  |  |  |  |  |  |  |  |
| --- | --- | --- | --- | --- | --- | --- | --- | --- | --- | --- |
| AgeGp | 0-14 | 15-39 | 40-44 | 45-49 | 50-54 | 55-59 | 60-64 | 65-69 | 70-74 | >74 |
| M | 0 | 0 | 0 | 0 | 0 | 0 | 0 | 0 | 0 | 0 |
| F | 0 | 14.5 | 43 | 72.7 | 108.1 | 110.6 | 131.2 | 134.1 | 165.7 | 190.5 |
| colorectal cancer | |  |  |  |  |  |  |  |  |  |
| AgeGp | 0-14 | 15-39 | 40-44 | 45-49 | 50-54 | 55-59 | 60-64 | 65-69 | 70-74 | >74 |
| M | 0 | 2.3 | 5.6 | 8.8 | 26.5 | 31.7 | 64 | 83 | 125.9 | 206.4 |
| F | 0 | 2.9 | 6.9 | 13.2 | 22 | 35.5 | 55.5 | 84.4 | 137.1 | 259.7 |
| coronary heart disease | | |  |  |  |  |  |  |  |  |
| AgeGp | 0-19 | 20-24 | 25-44 | 45-49 | 50-59 | 60-64 | >64 |  |  |  |
| M | 0 | 5.76 | 26.01 | 92.4 | 209.42 | 398.26 | 518.37 |  |  |  |
| F | 0 | 7.93 | 28.8 | 98.9 | 222.2 | 419 | 556.48 |  |  |  |
| diabetes |  |  |  |  |  |  |  |  |  |  |
| AgeGp | 0-19 | 20-24 | 25-44 | 45-49 | 50-59 | 60-64 | >64 |  |  |  |
| M | 0 | 21.35 | 155.1 | 475.56 | 2801.91 | 5272.95 | 562.76 |  |  |  |
| F | 0 | 31.63 | 193.63 | 902.64 | 587.52 | 740.18 | 917.29 |  |  |  |
| endometrial cancer | | |  |  |  |  |  |  |  |  |
| AgeGp | 0-14 | 15-39 | 40-44 | 45-49 | 50-54 | 55-59 | 60-64 | 65-69 | 70-74 | >74 |
| M | 0 | 0 | 0 | 0 | 0 | 0 | 0 | 0 | 0 | 0 |
| F | 0 | 1.6 | 4.8 | 16.7 | 37.3 | 55.2 | 63.1 | 73.4 | 83.4 | 105.7 |
| hypertension | |  |  |  |  |  |  |  |  |  |
| AgeGp | 0-19 | 20-24 | 25-44 | 45-49 | 50-59 | 60-64 | >64 |  |  |  |
| M | 0 | 33.83 | 230.11 | 631.65 | 2131.15 | 4001.47 | 1161.66 |  |  |  |
| F | 0 | 36.64 | 330.79 | 1308.15 | 1097.06 | 1382.08 | 1965.48 |  |  |  |
| kidney cancer | |  |  |  |  |  |  |  |  |  |
| AgeGp | 0-14 | 15-39 | 40-44 | 45-49 | 50-54 | 55-59 | 60-64 | 65-69 | 70-74 | >74 |
| M | 0.6 | 0.5 | 1.7 | 2.6 | 8.3 | 12.3 | 17.5 | 17.7 | 16.4 | 22.1 |
| F | 0.8 | 0.4 | 0.9 | 0.9 | 2.4 | 4.2 | 4.7 | 8.8 | 8.8 | 11 |
| liver cancer | |  |  |  |  |  |  |  |  |  |
| AgeGp | 0-14 | 15-39 | 40-44 | 45-49 | 50-54 | 55-59 | 60-64 | 65-69 | 70-74 | >74 |
| M | 0.1 | 0.3 | 0.7 | 2.6 | 5.7 | 10.9 | 16.8 | 24.2 | 38.4 | 61.2 |
| F | 0.1 | 0.4 | 1.1 | 2.3 | 5.5 | 10 | 11.9 | 17.1 | 32.7 | 60 |
| oesophageal cancer | | |  |  |  |  |  |  |  |  |
| AgeGp | 0-14 | 15-39 | 40-44 | 45-49 | 50-54 | 55-59 | 60-64 | 65-69 | 70-74 | >74 |
| M | 0 | 0.4 | 2.8 | 8.8 | 17.5 | 24.9 | 26.3 | 31.7 | 44.1 | 52.9 |
| F | 0 | 0 | 0.7 | 0.5 | 3.4 | 3.5 | 5 | 5.7 | 9.3 | 15.6 |
| osteoarthritis | |  |  |  |  |  |  |  |  |  |
| AgeGp | 0-9 | Oct-19 | 20-29 | 30-39 | 40-49 | 50-59 | 60-69 | 70-79 | 80-89 | >89 |
| M | 55.79 | 148.37 | 342.36 | 653.43 | 1144.98 | 1699.12 | 1791.07 | 1610.38 | 1466.83 | 1042.73 |
| F | 45.09 | 187.01 | 380.01 | 835.06 | 1696.47 | 2722.91 | 2473.07 | 2006.15 | 1840.18 | 923.28 |
| pancreatic cancer | |  |  |  |  |  |  |  |  |  |
| AgeGp | 0-14 | 15-39 | 40-44 | 45-49 | 50-54 | 55-59 | 60-64 | 65-69 | 70-74 | >74 |
| M | 0 | 0.3 | 2 | 4.8 | 8 | 11.9 | 19 | 28 | 41.5 | 64.9 |
| F | 0 | 0.3 | 1.3 | 3.3 | 6.1 | 8.1 | 14.4 | 20.2 | 36.8 | 59.6 |
| stroke |  |  |  |  |  |  |  |  |  |  |
| AgeGp | 0-24 | 25-34 | 35-44 | 45-54 | 55-64 | 65-74 | 75-84 | >84 |  |  |
| M | 8.38 | 9.65 | 34.92 | 133.4 | 463.82 | 546.42 | 1259.16 | 1754.19 |  |  |
| F | 1.69 | 4.61 | 19.18 | 112.99 | 287.31 | 641.81 | 1433.51 | 1565.28 |  |  |

Cuba mortality per 100,000

| breast cancer | |  |  |  |  |  |  |  |  |  |
| --- | --- | --- | --- | --- | --- | --- | --- | --- | --- | --- |
| AgeGp | 0-14 | 15-39 | 40-44 | 45-49 | 50-54 | 55-59 | 60-64 | 65-69 | 70-74 | >74 |
| M | 0 | 0 | 0 | 0 | 0 | 0 | 0 | 0 | 0 | 0 |
| F | 0 | 3 | 15.4 | 21.6 | 37.6 | 43.9 | 56.6 | 61.1 | 81.1 | 142.3 |
| colorectal cancer | |  |  |  |  |  |  |  |  |  |
| AgeGp | 0-14 | 15-39 | 40-44 | 45-49 | 50-54 | 55-59 | 60-64 | 65-69 | 70-74 | >74 |
| M | 0 | 0.8 | 3.4 | 4.5 | 14.7 | 18.4 | 38.5 | 51.7 | 83.7 | 182.3 |
| F | 0 | 0.9 | 4.6 | 6.7 | 12.2 | 20.6 | 33.5 | 52.8 | 91 | 235.2 |
| coronary heart disease | | |  |  |  |  |  |  |  |  |
| AgeGp | 0-14 | 15-29 | 30-44 | 45-59 | 60-69 | 70-79 | >79 |  |  |  |
| M | 0 | 5.33 | 23.97 | 79.88 | 320.75 | 831.94 | 1703.96 |  |  |  |
| F | 0.1 | 2.39 | 13.56 | 31.91 | 193.98 | 468.14 | 1810.47 |  |  |  |
| diabetes |  |  |  |  |  |  |  |  |  |  |
| non terminal | |  |  |  |  |  |  |  |  |  |
| endometrial cancer | | |  |  |  |  |  |  |  |  |
| AgeGp | 0-14 | 15-39 | 40-44 | 45-49 | 50-54 | 55-59 | 60-64 | 65-69 | 70-74 | >74 |
| M | 0 | 0 | 0 | 0 | 0 | 0 | 0 | 0 | 0 | 0 |
| F | 0 | 0.9 | 3 | 5.3 | 10.1 | 15.2 | 19.1 | 25.9 | 36.8 | 62.4 |
| hypertension | |  |  |  |  |  |  |  |  |  |
| non terminal | |  |  |  |  |  |  |  |  |  |
| kidney cancer | |  |  |  |  |  |  |  |  |  |
| AgeGp | 0-14 | 15-39 | 40-44 | 45-49 | 50-54 | 55-59 | 60-64 | 65-69 | 70-74 | >74 |
| M | 0.2 | 0.3 | 0.7 | 1.2 | 3.8 | 6.1 | 9.5 | 10.3 | 10.7 | 18.7 |
| F | 0.2 | 0.3 | 0.4 | 0.5 | 1.2 | 2.3 | 2.9 | 5.7 | 6.4 | 11 |
| liver cancer | |  |  |  |  |  |  |  |  |  |
| AgeGp | 0-14 | 15-39 | 40-44 | 45-49 | 50-54 | 55-59 | 60-64 | 65-69 | 70-74 | >74 |
| M | 0 | 0.2 | 0.9 | 2.4 | 5.7 | 10.9 | 16.8 | 24.2 | 37.8 | 59.5 |
| F | 0.1 | 0.2 | 1.1 | 2.1 | 5.5 | 10 | 11.9 | 17.1 | 32.1 | 58.5 |
| oesophageal cancer | | |  |  |  |  |  |  |  |  |
| AgeGp | 0-14 | 15-39 | 40-44 | 45-49 | 50-54 | 55-59 | 60-64 | 65-69 | 70-74 | >74 |
| M | 0 | 0.2 | 2.6 | 6.7 | 13.7 | 20.4 | 22.5 | 27.5 | 37.8 | 50.4 |
| F | 0 | 0 | 0.7 | 0.5 | 2.7 | 3.2 | 4.7 | 5.3 | 8.8 | 16.3 |
| osteoarthritis | |  |  |  |  |  |  |  |  |  |
| non terminal | |  |  |  |  |  |  |  |  |  |
| pancreatic cancer | |  |  |  |  |  |  |  |  |  |
| AgeGp | 0-14 | 15-39 | 40-44 | 45-49 | 50-54 | 55-59 | 60-64 | 65-69 | 70-74 | >74 |
| M | 0 | 0.3 | 1.9 | 4.5 | 7.7 | 11.2 | 18.3 | 27 | 40.3 | 64.1 |
| F | 0 | 0.1 | 1.5 | 3 | 5.8 | 7.7 | 13.7 | 19.3 | 35.6 | 58.9 |
| stroke |  |  |  |  |  |  |  |  |  |  |
| AgeGp | 0-14 | 15-29 | 30-44 | 45-59 | 60-69 | 70-79 | >79 |  |  |  |
| M | 0 | 5.33 | 23.97 | 79.88 | 320.75 | 831.94 | 1703.96 |  |  |  |
| F | 0.1 | 2.39 | 13.56 | 31.91 | 193.98 | 468.14 | 1810.47 |  |  |  |

Nicaragua incidence per 100,000

| breast cancer | |  |  |  |  |  |  |  |  |  |
| --- | --- | --- | --- | --- | --- | --- | --- | --- | --- | --- |
| AgeGp | 0-14 | 15-39 | 40-44 | 45-49 | 50-54 | 55-59 | 60-64 | 65-69 | 70-74 | >74 |
| F | 0 | 5.7 | 43.6 | 67 | 73.6 | 56.9 | 68.7 | 79.1 | 84.7 | 69 |
| colorectal cancer | |  |  |  |  |  |  |  |  |  |
| AgeGp | 0-14 | 15-39 | 40-44 | 45-49 | 50-54 | 55-59 | 60-64 | 65-69 | 70-74 | >74 |
| F | 0 | 1.5 | 8.6 | 9.8 | 9.6 | 19.4 | 27 | 35.2 | 58.4 | 81.7 |
| coronary heart disease | | |  |  |  |  |  |  |  |  |
| AgeGp | 0-19 | 20-24 | 25-44 | 45-49 | 50-59 | 60-64 | >64 |  |  |  |
| F | 0 | 7.03 | 25.53 | 87.69 | 197 | 371.49 | 493.38 |  |  |  |
| diabetes |  |  |  |  |  |  |  |  |  |  |
| AgeGp | 0-19 | 20-24 | 25-44 | 45-49 | 50-59 | 60-64 | >64 |  |  |  |
| F | 0 | 33.3 | 288.42 | 1256.54 | 746 | 914.64 | 1486.13 |  |  |  |
| endometrial cancer | | |  |  |  |  |  |  |  |  |
| AgeGp | 0-14 | 15-39 | 40-44 | 45-49 | 50-54 | 55-59 | 60-64 | 65-69 | 70-74 | >74 |
| F | 0 | 1 | 7.1 | 5.7 | 10.5 | 13.9 | 18.7 | 26.4 | 17.5 | 12.7 |
| hypertension | |  |  |  |  |  |  |  |  |  |
| AgeGp | 0-19 | 20-24 | 25-44 | 45-49 | 50-59 | 60-64 | >64 |  |  |  |
| F | 0 | 38.78 | 395.97 | 1519.43 | 1233.13 | 1543.07 | 2699.25 |  |  |  |
| kidney cancer | |  |  |  |  |  |  |  |  |  |
| AgeGp | 0-14 | 15-39 | 40-44 | 45-49 | 50-54 | 55-59 | 60-64 | 65-69 | 70-74 | >74 |
| F | 0.1 | 0 | 0.7 | 2.5 | 2.9 | 6.9 | 8.3 | 13.2 | 8.8 | 12.7 |
| liver cancer | |  |  |  |  |  |  |  |  |  |
| AgeGp | 0-14 | 15-39 | 40-44 | 45-49 | 50-54 | 55-59 | 60-64 | 65-69 | 70-74 | >74 |
| F | 0.3 | 0.9 | 5 | 8.2 | 12.4 | 25 | 41.6 | 52.7 | 67.1 | 99.9 |
| oesophageal cancer | | |  |  |  |  |  |  |  |  |
| AgeGp | 0-44 | 45-49 | 50-54 | 55-59 | 60-64 | 65-69 | 70-74 | >74 |  |  |
| F | 0 | 0 | 0 | 4.2 | 2.1 | 0 | 2.9 | 9.1 |  |  |
| osteoarthritis | |  |  |  |  |  |  |  |  |  |
| AgeGp | 0-9 | Oct-19 | 20-29 | 30-39 | 40-49 | 50-59 | 60-69 | 70-79 | 80-89 | >89 |
| F | 45.09 | 182.02 | 399.88 | 932.53 | 1846.92 | 2912.53 | 2692.5 | 2350.67 | 2303.19 | 1155.59 |
| pancreatic cancer | |  |  |  |  |  |  |  |  |  |
| AgeGp | 0-14 | 15-39 | 40-44 | 45-49 | 50-54 | 55-59 | 60-64 | 65-69 | 70-74 | >74 |
| F | 0 | 0 | 0.7 | 2.5 | 2.9 | 13.9 | 20.8 | 17.6 | 23.4 | 43.6 |
| stroke |  |  |  |  |  |  |  |  |  |  |
| AgeGp | 0-24 | 25-34 | 35-44 | 45-54 | 55-64 | 65-74 | 75-84 | >84 |  |  |
| F | 1.47 | 4.01 | 16.69 | 98.3 | 249.98 | 558.4 | 1247.22 | 1361.86 |  |  |

Nicaragua mortality per 100,000

| breast cancer | |  |  |  |  |  |  |  |  |  |
| --- | --- | --- | --- | --- | --- | --- | --- | --- | --- | --- |
| AgeGp | 0-14 | 15-39 | 40-44 | 45-49 | 50-54 | 55-59 | 60-64 | 65-69 | 70-74 | >74 |
| F | 0 | 1.4 | 12.9 | 20.4 | 28.7 | 25 | 29.1 | 30.8 | 35 | 45.4 |
| colorectal cancer | |  |  |  |  |  |  |  |  |  |
| AgeGp | 0-14 | 15-39 | 40-44 | 45-49 | 50-54 | 55-59 | 60-64 | 65-69 | 70-74 | >74 |
| F | 0 | 0.8 | 2.9 | 5.7 | 5.7 | 11.1 | 14.6 | 19.8 | 35 | 69 |
| coronary heart disease | | |  |  |  |  |  |  |  |  |
| AgeGp | 0-14 | 15-29 | 30-44 | 45-59 | 60-69 | 70-79 | >79 |  |  |  |
| F | 0 | 4.18 | 10.04 | 33.47 | 150.42 | 376.04 | 1479.09 |  |  |  |
| diabetes |  |  |  |  |  |  |  |  |  |  |
| non terminal | |  |  |  |  |  |  |  |  |  |
| endometrial cancer | | |  |  |  |  |  |  |  |  |
| AgeGp | 0-14 | 15-39 | 40-44 | 45-49 | 50-54 | 55-59 | 60-64 | 65-69 | 70-74 | >74 |
| F | 0 | 0.5 | 0.7 | 1.6 | 2.9 | 4.2 | 6.2 | 11 | 8.8 | 10.9 |
| hypertension | |  |  |  |  |  |  |  |  |  |
| non terminal | |  |  |  |  |  |  |  |  |  |
| kidney cancer | |  |  |  |  |  |  |  |  |  |
| AgeGp | 0-14 | 15-39 | 40-44 | 45-49 | 50-54 | 55-59 | 60-64 | 65-69 | 70-74 | >74 |
| F | 0.1 | 0 | 0 | 0.8 | 1 | 2.8 | 4.2 | 6.6 | 5.8 | 10.9 |
| liver cancer | |  |  |  |  |  |  |  |  |  |
| AgeGp | 0-14 | 15-39 | 40-44 | 45-49 | 50-54 | 55-59 | 60-64 | 65-69 | 70-74 | >74 |
| F | 0.1 | 1.2 | 3.6 | 8.2 | 12.4 | 25 | 41.6 | 52.7 | 67.1 | 99.9 |
| oesophageal cancer | | |  |  |  |  |  |  |  |  |
| AgeGp | 0-44 | 45-49 | 50-54 | 55-59 | 60-64 | 65-69 | 70-74 | >74 |  |  |
| F | 0 | 0 | 0 | 2.8 | 2.1 | 0 | 2.9 | 9.1 |  |  |
| osteoarthritis | |  |  |  |  |  |  |  |  |  |
| non terminal | |  |  |  |  |  |  |  |  |  |
| pancreatic cancer | |  |  |  |  |  |  |  |  |  |
| AgeGp | 0-14 | 15-39 | 40-44 | 45-49 | 50-54 | 55-59 | 60-64 | 65-69 | 70-74 | >74 |
| F | 0 | 0 | 1.4 | 2.5 | 2.9 | 13.9 | 20.8 | 17.6 | 23.4 | 43.6 |
| stroke |  |  |  |  |  |  |  |  |  |  |
| AgeGp | 0-14 | 15-29 | 30-44 | 45-59 | 60-69 | 70-79 | >79 |  |  |  |
| F | 0.21 | 2.73 | 5.46 | 20.48 | 105.5 | 236.69 | 811.51 |  |  |  |

Panama incidence per 100,000

| breast cancer | |  |  |  |  |  |  |  |  |  |
| --- | --- | --- | --- | --- | --- | --- | --- | --- | --- | --- |
| AgeGp | 0-14 | 15-39 | 40-44 | 45-49 | 50-54 | 55-59 | 60-64 | 65-69 | 70-74 | >74 |
| M | 0 | 0 | 0 | 0 | 0 | 0 | 0 | 0 | 0 | 0 |
| F | 0 | 8.2 | 41.9 | 59.1 | 56.7 | 87.9 | 106.1 | 123 | 148.8 | 142.8 |
| colorectal cancer | |  |  |  |  |  |  |  |  |  |
| AgeGp | 0-14 | 15-39 | 40-44 | 45-49 | 50-54 | 55-59 | 60-64 | 65-69 | 70-74 | >74 |
| M | 0 | 1 | 3.5 | 10.7 | 14.4 | 32.3 | 38.2 | 68.3 | 79.9 | 153.4 |
| F | 0 | 1 | 4.5 | 12.7 | 19.3 | 33.6 | 36 | 64 | 86.5 | 147.2 |
| coronary heart disease | | |  |  |  |  |  |  |  |  |
| AgeGp | 0-19 | 20-24 | 25-44 | 45-49 | 50-59 | 60-64 | >64 |  |  |  |
| M | 0 | 3.59 | 16.21 | 57.6 | 130.54 | 248.26 | 323.14 |  |  |  |
| F | 0 | 3.83 | 13.91 | 47.78 | 107.35 | 202.44 | 268.86 |  |  |  |
| diabetes |  |  |  |  |  |  |  |  |  |  |
| AgeGp | 0-19 | 20-24 | 25-44 | 45-49 | 50-59 | 60-64 | >64 |  |  |  |
| M | 0 | 23.5 | 203.87 | 647.48 | 3355.6 | 6765.51 | 681.37 |  |  |  |
| F | 0 | 35.05 | 258.48 | 1125.08 | 673.86 | 829.12 | 974.48 |  |  |  |
| endometrial cancer | | |  |  |  |  |  |  |  |  |
| AgeGp | 0-14 | 15-39 | 40-44 | 45-49 | 50-54 | 55-59 | 60-64 | 65-69 | 70-74 | >74 |
| M | 0 | 0 | 0 | 0 | 0 | 0 | 0 | 0 | 0 | 0 |
| F | 0 | 0.4 | 2.7 | 4.2 | 19.3 | 11.2 | 46 | 41 | 20.8 | 29 |
| hypertension | |  |  |  |  |  |  |  |  |  |
| AgeGp | 0-19 | 20-24 | 25-44 | 45-49 | 50-59 | 60-64 | >64 |  |  |  |
| M | 0 | 39.23 | 264.39 | 732.65 | 2375.31 | 4587.59 | 1291.54 |  |  |  |
| F | 0 | 42.91 | 380.72 | 1440.85 | 1173.63 | 1460.1 | 2084.96 |  |  |  |
| kidney cancer | |  |  |  |  |  |  |  |  |  |
| AgeGp | 0-14 | 15-39 | 40-44 | 45-49 | 50-54 | 55-59 | 60-64 | 65-69 | 70-74 | >74 |
| M | 0.2 | 0.3 | 0.9 | 6.4 | 9.2 | 6.5 | 8 | 21 | 32.7 | 23.4 |
| F | 0.2 | 0 | 0.9 | 0 | 3.9 | 14.4 | 12 | 5.1 | 17.3 | 15.6 |
| liver cancer | |  |  |  |  |  |  |  |  |  |
| AgeGp | 0-14 | 15-39 | 40-44 | 45-49 | 50-54 | 55-59 | 60-64 | 65-69 | 70-74 | >74 |
| M | 0 | 0.4 | 1.8 | 5.3 | 1.3 | 8.1 | 14.1 | 28.9 | 47.2 | 49.4 |
| F | 0.2 | 0.6 | 1.8 | 0 | 5.2 | 8 | 10 | 23.1 | 31.1 | 53.5 |
| oesophageal cancer | | |  |  |  |  |  |  |  |  |
| AgeGp | 0-44 | 45-49 | 50-54 | 55-59 | 60-64 | 65-69 | 70-74 | >74 |  |  |
| M | 0 | 1.1 | 5.2 | 14.5 | 12.1 | 10.5 | 25.4 | 28.6 |  |  |
| F | 0 | 1.1 | 1.3 | 1.6 | 6 | 10.2 | 0 | 11.2 |  |  |
| osteoarthritis | |  |  |  |  |  |  |  |  |  |
| AgeGp | 0-9 | Oct-19 | 20-29 | 30-39 | 40-49 | 50-59 | 60-69 | 70-79 | 80-89 | >89 |
| M | 55.79 | 148.37 | 379.1 | 716.75 | 1233.96 | 1866.64 | 1963.21 | 1757.78 | 1584.35 | 1126.27 |
| F | 45.09 | 187.01 | 414.63 | 896 | 1771.96 | 2806.18 | 2525.96 | 2083.7 | 1959.98 | 983.39 |
| pancreatic cancer | |  |  |  |  |  |  |  |  |  |
| AgeGp | 0-39 | 40-44 | 45-49 | 50-54 | 55-59 | 60-64 | 65-69 | 70-74 | >74 |  |
| M | 0 | 0.9 | 2.1 | 6.5 | 8.1 | 18.1 | 18.4 | 29 | 41.6 |  |
| F | 0 | 0.9 | 1.1 | 3.9 | 8 | 10 | 23.1 | 34.6 | 75.8 |  |
| stroke |  |  |  |  |  |  |  |  |  |  |
| AgeGp | 0-24 | 25-34 | 35-44 | 45-54 | 55-64 | 65-74 | 75-84 | >84 |  |  |
| M | 7.86 | 9.05 | 32.77 | 125.19 | 435.26 | 512.78 | 1181.65 | 1646.2 |  |  |
| F | 1.24 | 3.38 | 14.07 | 82.9 | 210.81 | 470.92 | 1051.83 | 1148.51 |  |  |

Panama mortality per 100,000

| breast cancer | |  |  |  |  |  |  |  |  |  |
| --- | --- | --- | --- | --- | --- | --- | --- | --- | --- | --- |
| AgeGp | 0-14 | 15-39 | 40-44 | 45-49 | 50-54 | 55-59 | 60-64 | 65-69 | 70-74 | >74 |
| M | 0 | 0 | 0 | 0 | 0 | 0 | 0 | 0 | 0 | 0 |
| F | 0 | 1.8 | 13.4 | 18 | 21.9 | 38.4 | 44 | 48.7 | 62.3 | 100.4 |
| colorectal cancer | |  |  |  |  |  |  |  |  |  |
| AgeGp | 0-14 | 15-39 | 40-44 | 45-49 | 50-54 | 55-59 | 60-64 | 65-69 | 70-74 | >74 |
| M | 0 | 0.1 | 3.5 | 6.4 | 9.2 | 19.4 | 22.1 | 39.4 | 50.8 | 140.4 |
| F | 0 | 0.4 | 2.7 | 7.4 | 11.6 | 19.2 | 20 | 35.9 | 51.9 | 131.6 |
| coronary heart disease | | |  |  |  |  |  |  |  |  |
| AgeGp | 0-14 | 15-29 | 30-44 | 45-59 | 60-60 | 61-69 | 70-79 | >79 |  |  |
| M | 0 | 2.81 | 15.93 | 37.48 | 18.74 | 145.49 | 339.47 | 1185.73 |  |  |
| F | 0 | 2.87 | 6.46 | 16.5 | 130.69 | 130.69 | 264.74 | 683.64 |  |  |
| diabetes |  |  |  |  |  |  |  |  |  |  |
| non terminal | |  |  |  |  |  |  |  |  |  |
| endometrial cancer | | |  |  |  |  |  |  |  |  |
| AgeGp | 0-14 | 15-39 | 40-44 | 45-49 | 50-54 | 55-59 | 60-64 | 65-69 | 70-74 | >74 |
| M | 0 | 0 | 0 | 0 | 0 | 0 | 0 | 0 | 0 | 0 |
| F | 0 | 0.1 | 0.9 | 1.1 | 5.2 | 3.2 | 16 | 17.9 | 10.4 | 24.5 |
| hypertension | |  |  |  |  |  |  |  |  |  |
| non terminal | |  |  |  |  |  |  |  |  |  |
| kidney cancer | |  |  |  |  |  |  |  |  |  |
| AgeGp | 0-14 | 15-44 | 45-49 | 50-54 | 55-59 | 60-64 | 65-69 | 70-74 | >74 |  |
| M | 0.4 | 0 | 2.1 | 3.9 | 3.2 | 4 | 13.1 | 21.8 | 23.4 |  |
| F | 0.2 | 0 | 0 | 1.3 | 6.4 | 6 | 2.6 | 10.4 | 13.4 |  |
| kidney cancer | |  |  |  |  |  |  |  |  |  |
| AgeGp | 0-14 | 15-44 | 45-49 | 50-54 | 55-59 | 60-64 | 65-69 | 70-74 | >74 |  |
| M | 0.4 | 0 | 2.1 | 3.9 | 3.2 | 4 | 13.1 | 21.8 | 23.4 |  |
| F | 0.2 | 0 | 0 | 1.3 | 6.4 | 6 | 2.6 | 10.4 | 13.4 |  |
| liver cancer | |  |  |  |  |  |  |  |  |  |
| AgeGp | 0-14 | 15-39 | 40-44 | 45-49 | 50-54 | 55-59 | 60-64 | 65-69 | 70-74 | >74 |
| M | 0 | 0.7 | 0 | 5.3 | 1.3 | 8.1 | 14.1 | 28.9 | 47.2 | 49.4 |
| F | 0.2 | 0.3 | 1.8 | 0 | 5.2 | 8 | 10 | 23.1 | 31.1 | 53.5 |
| oesophageal cancer | | |  |  |  |  |  |  |  |  |
| AgeGp | 0-44 | 45-49 | 50-54 | 55-59 | 60-64 | 65-69 | 70-74 | >74 |  |  |
| M | 0 | 1.1 | 3.9 | 11.3 | 10 | 7.9 | 21.8 | 28.6 |  |  |
| F | 0 | 1.1 | 1.3 | 1.6 | 4 | 7.7 | 0 | 11.2 |  |  |
| osteoarthritis | |  |  |  |  |  |  |  |  |  |
| non terminal | |  |  |  |  |  |  |  |  |  |
| pancreatic cancer | |  |  |  |  |  |  |  |  |  |
| AgeGp | 0-14 | 15-39 | 40-44 | 45-49 | 50-54 | 55-59 | 60-64 | 65-69 | 70-74 | >74 |
| M | 0 | 0 | 0.9 | 2.1 | 6.5 | 8.1 | 18.1 | 18.4 | 25.4 | 41.6 |
| F | 0 | 0.3 | 0 | 1.1 | 3.9 | 8 | 10 | 23.1 | 34.6 | 75.8 |
| stroke |  |  |  |  |  |  |  |  |  |  |
| AgeGp | 0-14 | 15-29 | 30-44 | 45-59 | 60-69 | 70-79 | >79 |  |  |  |
| M | 0.24 | 3.24 | 7.29 | 26.72 | 110.76 | 249.2 | 833.19 |  |  |  |
| F | 0.55 | 2.57 | 5.14 | 14.4 | 106.88 | 216.91 | 660.15 |  |  |  |

Peru incidence per 100,000

| breast cancer | |  |  |  |  |  |  |  |  |  |
| --- | --- | --- | --- | --- | --- | --- | --- | --- | --- | --- |
| AgeGp | 0-14 | 15-39 | 40-44 | 45-49 | 50-54 | 55-59 | 60-64 | 65-69 | 70-74 | >74 |
| F | 0 | 8.8 | 60.9 | 94.5 | 105.4 | 93.9 | 107.5 | 110.1 | 118.1 | 120.4 |
| colorectal cancer | |  |  |  |  |  |  |  |  |  |
| AgeGp | 0-14 | 15-39 | 40-44 | 45-49 | 50-54 | 55-59 | 60-64 | 65-69 | 70-74 | >74 |
| F | 0 | 1.9 | 4.8 | 10.1 | 14.8 | 19.2 | 28.5 | 46.5 | 56.6 | 102.3 |
| coronary heart disease | | |  |  |  |  |  |  |  |  |
| AgeGp | 0-19 | 20-24 | 25-44 | 45-49 | 50-59 | 60-64 | >64 |  |  |  |
| F | 0 | 2.93 | 10.64 | 36.55 | 82.12 | 154.85 | 205.66 |  |  |  |
| diabetes |  |  |  |  |  |  |  |  |  |  |
| AgeGp | 0-19 | 20-24 | 25-44 | 45-49 | 50-59 | 60-64 | >64 |  |  |  |
| F | 0 | 31.53 | 246.77 | 1077.03 | 589.26 | 732.23 | 1203.88 |  |  |  |
| endometrial cancer | | |  |  |  |  |  |  |  |  |
| AgeGp | 0-14 | 15-39 | 40-44 | 45-49 | 50-54 | 55-59 | 60-64 | 65-69 | 70-74 | >74 |
| F | 0 | 0.6 | 8.7 | 18.5 | 13.8 | 12.9 | 12.7 | 14.2 | 9.4 | 14.6 |
| hypertension | |  |  |  |  |  |  |  |  |  |
| AgeGp | 0-19 | 20-24 | 25-44 | 45-49 | 50-59 | 60-64 | >64 |  |  |  |
| F | 0 | 35.77 | 370.21 | 1412.82 | 1097.25 | 1373.04 | 2401.82 |  |  |  |
| kidney cancer | |  |  |  |  |  |  |  |  |  |
| AgeGp | 0-14 | 15-39 | 40-44 | 45-49 | 50-54 | 55-59 | 60-64 | 65-69 | 70-74 | >74 |
| F | 0.5 | 0.2 | 1.4 | 1.6 | 4 | 2.5 | 4.7 | 12.9 | 9.4 | 15.2 |
| liver cancer | |  |  |  |  |  |  |  |  |  |
| AgeGp | 0-14 | 15-39 | 40-44 | 45-49 | 50-54 | 55-59 | 60-64 | 65-69 | 70-74 | >74 |
| F | 0.4 | 1.5 | 1 | 7.4 | 11.3 | 17.6 | 25.9 | 46.5 | 59.5 | 92.7 |
| oesophageal cancer | | |  |  |  |  |  |  |  |  |
| AgeGp | 0-14 | 15-39 | 40-44 | 45-49 | 50-54 | 55-59 | 60-64 | 65-69 | 70-74 | >74 |
| F | 0 | 0.1 | 0 | 1.4 | 0.7 | 0.6 | 1.6 | 3.6 | 3.7 | 12.2 |
| osteoarthritis | |  |  |  |  |  |  |  |  |  |
| AgeGp | 0-9 | Oct-19 | 20-29 | 30-39 | 40-49 | 50-59 | 60-69 | 70-79 | 80-89 | >89 |
| F | 45.09 | 182.94 | 378.6 | 880.3 | 1833.22 | 2720.35 | 2514.83 | 2195.56 | 2151.21 | 1079.34 |
| pancreatic cancer | |  |  |  |  |  |  |  |  |  |
| AgeGp | 0-14 | 15-39 | 40-44 | 45-49 | 50-54 | 55-59 | 60-64 | 65-69 | 70-74 | >74 |
| F | 0 | 0.2 | 1.7 | 3 | 5 | 8.6 | 11.4 | 21.6 | 33.2 | 51.3 |
| stroke |  |  |  |  |  |  |  |  |  |  |
| AgeGp | 0-24 | 25-34 | 35-44 | 45-54 | 55-64 | 65-74 | 75-84 | >84 |  |  |
| F | 0.95 | 2.59 | 10.76 | 63.4 | 161.23 | 360.15 | 804.42 | 878.37 |  |  |

Peru mortality per 100,000

| breast cancer | |  |  |  |  |  |  |  |  |  |
| --- | --- | --- | --- | --- | --- | --- | --- | --- | --- | --- |
| AgeGp | 0-14 | 15-39 | 40-44 | 45-49 | 50-54 | 55-59 | 60-64 | 65-69 | 70-74 | >74 |
| F | 0 | 1.5 | 11.1 | 18.7 | 30.2 | 45.6 | 33.4 | 47.9 | 74.8 | 112.3 |
| colorectal cancer | |  |  |  |  |  |  |  |  |  |
| AgeGp | 0-14 | 15-39 | 40-44 | 45-49 | 50-54 | 55-59 | 60-64 | 65-69 | 70-74 | >74 |
| F | 0 | 0.7 | 3.9 | 5 | 10.7 | 17.5 | 27 | 35.4 | 50.7 | 135.7 |
| coronary heart disease | | |  |  |  |  |  |  |  |  |
| AgeGp | 0-14 | 15-29 | 30-44 | 45-59 | 60-69 | 70-79 | >79 |  |  |  |
| F | 0 | 2.32 | 5.8 | 14.49 | 110.45 | 276.13 | 1118.33 |  |  |  |
| diabetes |  |  |  |  |  |  |  |  |  |  |
| non terminal | |  |  |  |  |  |  |  |  |  |
| endometrial cancer | | |  |  |  |  |  |  |  |  |
| AgeGp | 0-39 | 40-49 | 50-54 | 55-59 | 60-64 | 65-69 | 70-74 | >74 |  |  |
| F | 0 | 0.7 | 1.8 | 4.7 | 6.4 | 8.3 | 16 | 28.1 |  |  |
| hypertension | |  |  |  |  |  |  |  |  |  |
| non terminal | |  |  |  |  |  |  |  |  |  |
| kidney cancer | |  |  |  |  |  |  |  |  |  |
| AgeGp | 0-14 | 15-39 | 40-44 | 45-49 | 50-54 | 55-59 | 60-64 | 65-69 | 70-74 | >74 |
| F | 0.2 | 0.1 | 0 | 0.7 | 0.9 | 4.7 | 3.2 | 6.3 | 10.7 | 12.5 |
| liver cancer | |  |  |  |  |  |  |  |  |  |
| AgeGp | 0-14 | 15-39 | 40-44 | 45-49 | 50-54 | 55-59 | 60-64 | 65-69 | 70-74 | >74 |
| F | 0 | 0.3 | 0.7 | 2.2 | 1.8 | 7 | 15.9 | 20.8 | 45.4 | 68.6 |
| oesophageal cancer | | |  |  |  |  |  |  |  |  |
| AgeGp | 0-44 | 45-49 | 50-54 | 55-59 | 60-64 | 65-69 | 70-74 | >74 |  |  |
| F | 0 | 0 | 0.9 | 1.2 | 1.6 | 2.1 | 2.7 | 15.6 |  |  |
| osteoarthritis | |  |  |  |  |  |  |  |  |  |
| non terminal | |  |  |  |  |  |  |  |  |  |
| pancreatic cancer | |  |  |  |  |  |  |  |  |  |
| AgeGp | 0-14 | 15-39 | 40-44 | 45-49 | 50-54 | 55-59 | 60-64 | 65-69 | 70-74 | >74 |
| F | 0 | 0.1 | 0 | 2.2 | 6.2 | 8.2 | 9.5 | 20.8 | 37.4 | 74.9 |
| stroke |  |  |  |  |  |  |  |  |  |  |
| AgeGp | 0-14 | 15-29 | 30-44 | 45-59 | 60-69 | 70-79 | >79 |  |  |  |
| F | 0.41 | 2.69 | 10.74 | 24.16 | 140.22 | 300.89 | 978.63 |  |  |  |

Uruguay incidence per 100,000

| breast cancer | |  |  |  |  |  |  |  |  |  |
| --- | --- | --- | --- | --- | --- | --- | --- | --- | --- | --- |
| AgeGp | 0-14 | 15-39 | 40-44 | 45-49 | 50-54 | 55-59 | 60-64 | 65-69 | 70-74 | >74 |
| M | 0 | 0 | 0 | 0 | 0 | 0 | 0 | 0 | 0 | 0 |
| F | 0 | 26.2 | 153.4 | 170.8 | 287.1 | 358.6 | 194.9 | 350 | 364.4 | 362 |
| colorectal cancer | |  |  |  |  |  |  |  |  |  |
| AgeGp | 0-14 | 15-39 | 40-44 | 45-49 | 50-54 | 55-59 | 60-64 | 65-69 | 70-74 | >74 |
| M | 0 | 2.3 | 10 | 19.1 | 41 | 82.6 | 131.7 | 214.3 | 329 | 321.3 |
| F | 0 | 3.3 | 21.8 | 25.3 | 47 | 49.4 | 102.4 | 114.8 | 158.5 | 268.1 |
| coronary heart disease | | |  |  |  |  |  |  |  |  |
| AgeGp | 0-19 | 20-24 | 25-44 | 45-49 | 50-59 | 60-64 | >64 |  |  |  |
| M | 0 | 4.66 | 21.04 | 74.73 | 169.37 | 322.1 | 419.24 |  |  |  |
| F | 0 | 3.9 | 14.17 | 48.65 | 109.29 | 206.09 | 273.71 |  |  |  |
| diabetes |  |  |  |  |  |  |  |  |  |  |
| AgeGp | 0-19 | 20-24 | 25-44 | 45-49 | 50-59 | 60-64 | >64 |  |  |  |
| M | 0 | 22.2 | 186.26 | 654.21 | 3879.45 | 8011.7 | 922.89 |  |  |  |
| F | 0 | 31.27 | 224.09 | 986.89 | 574.77 | 704.13 | 1046.85 |  |  |  |
| endometrial cancer | | |  |  |  |  |  |  |  |  |
| AgeGp | 0-14 | 15-39 | 40-44 | 45-59 | 60-64 | 65-69 | 70-74 | >74 |  |  |
| M | 0 | 0 | 0 | 0 | 0 | 0 | 0 | 0 |  |  |
| F | 0 | 0.3 | 2.8 | 0 | 25 | 24.9 | 26.7 | 61.2 |  |  |
| hypertension | |  |  |  |  |  |  |  |  |  |
| AgeGp | 0-19 | 20-24 | 25-44 | 45-49 | 50-59 | 60-64 | >64 |  |  |  |
| M | 0 | 35.97 | 253.92 | 744.79 | 2552.87 | 5033.07 | 1550.68 |  |  |  |
| F | 0 | 35.74 | 337.58 | 1339.45 | 1097.42 | 1378.07 | 2224.36 |  |  |  |
| kidney cancer | |  |  |  |  |  |  |  |  |  |
| AgeGp | 0-14 | 15-39 | 40-44 | 45-49 | 50-54 | 55-59 | 60-64 | 65-69 | 70-74 | >74 |
| M | 0.3 | 0.2 | 3 | 9 | 29.9 | 41.9 | 51.2 | 93.1 | 65.4 | 99.2 |
| F | 0.5 | 0.2 | 1.9 | 8.4 | 10.2 | 11.5 | 18.7 | 23.5 | 26.7 | 27.7 |
| liver cancer | |  |  |  |  |  |  |  |  |  |
| AgeGp | 0-44 | 45-49 | 50-54 | 55-59 | 60-64 | 65-69 | 70-74 | >74 |  |  |
| M | 0 | 1 | 5.5 | 8.9 | 19 | 22.8 | 16.9 | 26.4 |  |  |
| F | 0 | 0 | 1 | 2.3 | 2.5 | 1.4 | 14.8 | 8.5 |  |  |
| oesophageal cancer | | |  |  |  |  |  |  |  |  |
| AgeGp | 0-39 | 40-44 | 45-49 | 50-54 | 55-59 | 60-64 | 65-69 | 70-74 | >74 |  |
| M | 0 | 0 | 9 | 18.8 | 38.1 | 51.2 | 63.2 | 71.7 | 140.1 |  |
| F | 0 | 0.9 | 7.5 | 2 | 6.9 | 8.7 | 18 | 22.2 | 59 |  |
| osteoarthritis | |  |  |  |  |  |  |  |  |  |
| AgeGp | 0-9 | Oct-19 | 20-29 | 30-39 | 40-49 | 50-59 | 60-69 | 70-79 | 80-89 | >89 |
| M | 55.79 | 148.37 | 361.52 | 706.2 | 1257.99 | 1929.92 | 2090.8 | 1953.4 | 1819.55 | 1293.47 |
| F | 45.09 | 187.01 | 370.25 | 816.57 | 1678.4 | 2747.05 | 2550.95 | 2161.75 | 2058.73 | 1032.94 |
| pancreatic cancer | |  |  |  |  |  |  |  |  |  |
| AgeGp | 0-14 | 15-39 | 40-44 | 45-49 | 50-54 | 55-59 | 60-64 | 65-69 | 70-74 | >74 |
| M | 0 | 0 | 1 | 7 | 11.1 | 28 | 30.7 | 75.5 | 69.6 | 109.7 |
| F | 0 | 0.2 | 0.9 | 5.6 | 13.3 | 13.8 | 35 | 52.6 | 59.3 | 101.7 |
| stroke |  |  |  |  |  |  |  |  |  |  |
| AgeGp | 0-24 | 25-34 | 35-44 | 45-54 | 55-64 | 65-74 | 75-84 | >84 |  |  |
| M | 10.98 | 12.64 | 45.75 | 174.77 | 607.65 | 715.88 | 1649.64 | 2298.19 |  |  |
| F | 1.96 | 5.32 | 22.17 | 130.58 | 332.07 | 741.78 | 1656.79 | 1809.09 |  |  |

Uruguay mortality per 100,000

| breast cancer | |  |  |  |  |  |  |  |  |  |
| --- | --- | --- | --- | --- | --- | --- | --- | --- | --- | --- |
| AgeGp | 0-14 | 15-39 | 40-44 | 45-49 | 50-54 | 55-59 | 60-64 | 65-69 | 70-74 | >74 |
| M | 0 | 0 | 0 | 0 | 0 | 0 | 0 | 0 | 0 | 0 |
| F | 0 | 2.8 | 33.1 | 31 | 61.3 | 91.9 | 58.7 | 119 | 136.3 | 198.4 |
| colorectal cancer | |  |  |  |  |  |  |  |  |  |
| AgeGp | 0-14 | 15-39 | 40-44 | 45-49 | 50-54 | 55-59 | 60-64 | 65-69 | 70-74 | >74 |
| M | 0 | 0.7 | 6 | 8 | 18.8 | 40.7 | 70.3 | 123 | 208.8 | 263.1 |
| F | 0 | 1.8 | 5.7 | 10.3 | 20.4 | 23 | 51.2 | 62.2 | 94.8 | 218.4 |
| coronary heart disease | | |  |  |  |  |  |  |  |  |
| AgeGp | 0-14 | 15-29 | 30-44 | 45-59 | 60-69 | 70-79 | >79 |  |  |  |
| M | 0.29 | 7.48 | 22.44 | 65.45 | 217.71 | 495.88 | 1586.83 |  |  |  |
| F | 0.27 | 1.67 | 9.45 | 17.79 | 121.66 | 279.67 | 1006.82 |  |  |  |
| diabetes |  |  |  |  |  |  |  |  |  |  |
| non terminal | |  |  |  |  |  |  |  |  |  |
| endometrial cancer | |  |  |  |  |  |  |  |  |  |
| AgeGp | 0-39 | 40-44 | 45-59 | 60-64 | 65-69 | 70-74 | >74 |  |  |  |
| M | 0 | 0 | 0 | 0 | 0 | 0 | 0 |  |  |  |
| F | 0 | 0.9 | 0 | 10 | 11.1 | 13.3 | 48.4 |  |  |  |
| hypertension | |  |  |  |  |  |  |  |  |  |
| non terminal | |  |  |  |  |  |  |  |  |  |
| kidney cancer | |  |  |  |  |  |  |  |  |  |
| AgeGp | 0-14 | 15-39 | 40-44 | 45-49 | 50-54 | 55-59 | 60-64 | 65-69 | 70-74 | >74 |
| M | 0 | 0 | 2 | 4 | 14.4 | 21.6 | 27.8 | 54.5 | 42.2 | 76.7 |
| F | 0 | 0.2 | 0.9 | 3.8 | 5.1 | 5.7 | 10 | 13.8 | 17.8 | 21.3 |
| liver cancer | |  |  |  |  |  |  |  |  |  |
| AgeGp | 0-39 | 40-44 | 45-49 | 50-54 | 55-59 | 60-64 | 65-69 | 70-74 | >74 |  |
| M | 0 | 1 | 1 | 5.5 | 8.9 | 17.6 | 21.1 | 16.9 | 26.4 |  |
| F | 0 | 0.9 | 0 | 1 | 2.3 | 2.5 | 1.4 | 14.8 | 8.5 |  |
| oesophageal cancer | |  |  |  |  |  |  |  |  |  |
| AgeGp | 0-39 | 40-44 | 45-49 | 50-54 | 55-59 | 60-64 | 65-69 | 70-74 | >74 |  |
| M | 0 | 0 | 7 | 14.4 | 30.5 | 43.9 | 56.2 | 65.4 | 128.2 |  |
| F | 0 | 0.9 | 4.7 | 1 | 4.6 | 6.2 | 13.8 | 17.8 | 46.9 |  |
| osteoarthritis | |  |  |  |  |  |  |  |  |  |
| non terminal | |  |  |  |  |  |  |  |  |  |
| pancreatic cancer | |  |  |  |  |  |  |  |  |  |
| AgeGp | 0-14 | 15-39 | 40-44 | 45-49 | 50-54 | 55-59 | 60-64 | 65-69 | 70-74 | >74 |
| M | 0 | 0.2 | 0 | 6 | 10 | 26.7 | 29.3 | 73.8 | 69.6 | 107.1 |
| F | 0 | 0.2 | 0.9 | 4.7 | 12.3 | 12.6 | 32.5 | 49.8 | 57.8 | 98.2 |
| stroke |  |  |  |  |  |  |  |  |  |  |
| AgeGp | 0-14 | 15-29 | 30-44 | 45-59 | 60-69 | 70-79 | >79 |  |  |  |
| M | 0.29 | 7.48 | 22.44 | 65.45 | 217.71 | 495.88 | 1586.83 |  |  |  |
| F | 0.27 | 1.67 | 9.45 | 17.79 | 121.66 | 279.67 | 1006.82 |  |  |  |

Latin American survival data

| breast cancer |  |  |  |
| --- | --- | --- | --- |
| AgeGp | >14 |  |  |
| male Rate | 0 |  |  |
| female Rate | 0.0771 |  |  |
| male p(5 yr survival) | 1 |  |  |
| female p(5 yr survival) | 0.68 |  |  |
| colorectal cancer |  |  |  |
| AgeGp | >14 |  |  |
| male Rate | 0.1038 |  |  |
| female Rate | 0.0956 |  |  |
| male p(5 yr survival) | 0.595 |  |  |
| female p(5 yr survival) | 0.62 |  |  |
| coronary heart disease |  |  |  |
| AgeGp | 0-39 | 40-69 | >69 |
| male Rate | 0.0505 | 0.0298 | 0.1118 |
| female Rate | 0.0752 | 0.0481 | 0.1337 |
| male p1 | 0.18 | 0.11 | 0.27 |
| female p1 | 0.23 | 0.12 | 0.3 |
| male p(5 yr survival) | 0.67 | 0.79 | 0.4668 |
| female p(5 yr survival) | 0.57 | 0.726 | 0.4101 |
| endometrial cancer |  |  |  |
| AgeGp | >14 |  |  |
| male Rate | 0 |  |  |
| female Rate | 0.0484 |  |  |
| male p(5 yr survival) | 1 |  |  |
| female p(5 yr survival) | 0.785 |  |  |
| kidney cancer |  |  |  |
| AgeGp | >14 |  |  |
| male Rate | 0.0893 |  |  |
| female Rate | 0.0893 |  |  |
| male p(5 yr survival) | 0.64 |  |  |
| female p(5 yr survival) | 0.64 |  |  |
| liver cancer |  |  |  |
| AgeGp | >14 |  |  |
| male Rate | 0.8026 |  |  |
| female Rate | 0.8026 |  |  |
| male p(5 yr survival) | 0.0181 |  |  |
| female p(5 yr survival) | 0.0181 |  |  |
| oesophageal cancer |  |  |  |
| AgeGp | >14 |  |  |
| male Rate | 0.3568 |  |  |
| female Rate | 0.3568 |  |  |
| male p(5 yr survival) | 0.168 |  |  |
| female p(5 yr survival) | 0.168 |  |  |
|  |  |  |  |
| pancreatic cancer |  |  |  |
| AgeGp | >14 |  |  |
| male Rate | 0.5801 |  |  |
| female Rate | 0.5801 |  |  |
| male p(5 yr survival) | 0.055 |  |  |
| female p(5 yr survival) | 0.055 |  |  |
| stroke |  |  |  |
| AgeGp | 0-39 | 40-69 | >69 |
| male Rate | 0.0998 | 0.0565 | 0.1201 |
| female Rate | 0.1097 | 0.0628 | 0.1333 |
| male p1 | 0.21 | 0.16 | 0.24 |
| female p1 | 0.24 | 0.19 | 0.25 |
| male p(5 yr survival) | 0.53 | 0.6701 | 0.4701 |
| female p(5 yr survival) | 0.4901 | 0.6301 | 0.44 |
|  | | | |
