# Supplementary material for: High Rates of Obesity and Non-Communicable Diseases Predicted across Latin America
Source: PLoS One. 2012 Aug 13;7(8):e39589. doi: 10.1371/journal.pone.0039589 (PMC3418261; doi:10.1371/journal.pone.0039589)
Supplement: Table S4 — Percentage of overweight and obese males in Latin America projected to 2050. (DOCX) [file pone.0039589.s005.docx]

Table S5 Percentage of overweight and obese males in Latin America projected to 2050.

| Year | Argentina | Chile | Colombia | Costa  Rica | Cuba | Panama | Uruguay |
| --- | --- | --- | --- | --- | --- | --- | --- |
| 2010 | 43 | 67 | 54 | 63 | 58 | 63 | 62 |
| 2020 | 49 | 70 | 57 | 68 | 79 | 78 | 63 |
| 2030 | 53 | 72 | 62 | 70 | 88 | 86 | 66 |
| 2040 | 56 | 74 | 65 | 72 | 93 | 89 | 67 |
| 2050 | 58 | 74 | 68 | 72 | 94 | 92 | 68 |
