# Supplementary material for: High Rates of Obesity and Non-Communicable Diseases Predicted across Latin America
Source: PLoS One. 2012 Aug 13;7(8):e39589. doi: 10.1371/journal.pone.0039589 (PMC3418261; doi:10.1371/journal.pone.0039589)
Supplement: Table S5 — Percentage of overweight and obese females in Latin America projected to 2050. (DOCX) [file pone.0039589.s006.docx]

Table S6 Percentage of overweight and obese females in Latin America projected to 2050.

| Year | Argentina | Bolivia | Chile | Colombia | Costa  Rica | Cuba | Nicaragua | Panama | Peru | Uruguay |
| --- | --- | --- | --- | --- | --- | --- | --- | --- | --- | --- |
| 2010 | 24 | 60 | 66 | 56 | 61 | 67 | 64 | 69 | 66 | 64 |
| 2020 | 26 | 67 | 73 | 59 | 63 | 81 | 73 | 82 | 74 | 73 |
| 2030 | 29 | 73 | 79 | 63 | 64 | 85 | 80 | 88 | 81 | 80 |
| 2040 | 30 | 77 | 84 | 66 | 65 | 88 | 85 | 91 | 86 | 84 |
| 2050 | 30 | 81 | 87 | 70 | 65 | 89 | 88 | 94 | 88 | 86 |
