# Supplementary material for: High Rates of Obesity and Non-Communicable Diseases Predicted across Latin America
Source: PLoS One. 2012 Aug 13;7(8):e39589. doi: 10.1371/journal.pone.0039589 (PMC3418261; doi:10.1371/journal.pone.0039589)
Supplement: Table S6 — Cumulative incidence cases avoided per 100,000 of the population in 2010 with a 1% (scenario 1) and 5% (scenario 2) decrease in body mass index by 2030. (DOCX) [file pone.0039589.s007.docx]

Table S7 Cumulative incidence cases avoided per 100,000 of the population in 2010 with a 1% (scenario 1) and 5% (scenario 2) decrease in body mass index by 2030.

|  |  | Cancers | CHD+Stroke | Diabetes |
| --- | --- | --- | --- | --- |
| Scenario 1 | Argentina | 14 | 128 | 119 |
|  | Bolivia | 10 | 107 | 311 |
|  | Chile | 16 | 177 | 413 |
|  | Colombia | 14 | 189 | 423 |
|  | Cuba | 36 | 404 | 818 |
|  | Nicaragua | 13 | 202 | 497 |
|  | Panama | 39 | 178 | 435 |
|  | Peru | 29 | 99 | 375 |
|  | Uruguay | 19 | 242 | 480 |
| Scenario 2 | Argentina | 77 | 483 | 629 |
|  | Bolivia | 35 | 433 | 932 |
|  | Chile | 79 | 801 | 1332 |
|  | Colombia | 53 | 690 | 1124 |
|  | Cuba | 128 | 1451 | 2150 |
|  | Nicaragua | 39 | 662 | 1299 |
|  | Panama | 51 | 582 | 1354 |
|  | Peru | 73 | 621 | 1229 |
|  | Uruguay | 109 | 1057 | 1519 |
